# Tannic acid-assisted mechanical training transforms natural hydrogels into robust and bioactive membranes for guided bone regeneration

**DOI:** 10.1016/j.mtbio.2026.102863

**Published:** 2026-02-01

**Authors:** Jing Sun, Xi Wang, Xiaoxue Wang, Wenhui Yu, Yang Yu, Shaohua Ge, Zheqin Dong

**Affiliations:** Department of Periodontology & Prosthodontics & Additive Manufacturing, School and Hospital of Stomatology, Shandong University & Shandong Key Laboratory of Oral Tissue Regeneration & Shandong Engineering Research Center of Dental Materials and Oral Tissue Regeneration & Shandong Provincial Clinical Research Center for Oral Diseases, Jinan, Shandong, China

**Keywords:** Mechanical training, Tannic acid, Natural hydrogel, ROS scavenging, Anti-inflammatory, Guided bone regeneration

## Abstract

Guided bone regeneration (GBR) membranes are widely used for the treatment of bone defects. Natural hydrogels are promising candidates for GBR membranes owing to their excellent bioactivity and controllable degradability, but their clinical translation is restricted by inherent mechanical weakness. Inspired by tendon-strengthening mechanisms in athletes, we propose a tannic acid (TA)-assisted wet-stretching (TAWS) strategy to transform gelatin methacryloyl (GelMA) hydrogels into mechanically robust GBR membranes. During stretching, GelMA chains are directionally aligned while TA establishes multivalent hydrogen bonds between adjacent fibers, synergistically reinforcing the network. The resulting TA-trained (GHT) membranes achieved a 22.16-fold increase in Young's modulus and a 12.31-fold enhancement in toughness. In parallel, TAWS markedly slowed degradation kinetics and enhanced physiological stability, enabling GHT membranes to retain ∼80 % of their initial mass after 28 days in SBF. Beyond reinforcement, TA imparted potent ROS-scavenging and immunomodulatory activity. In vitro, GHT membranes enhanced stem cell survival, proliferation, and osteogenic differentiation under oxidative stress. In a mandibular defect model under elevated oxidative and inflammatory challenge, GHT reduced ROS levels (DHE fluorescence) to 53.76 % of the untreated ROS-upregulated group and increased bone volume fraction (BV/TV) by approximately 2.68-fold at 4 weeks and 2.21-fold at 8 weeks, outperforming the Bio-Gide® membrane. Collectively, TAWS provides a scalable platform to engineer multifunctional hydrogel membranes that integrate mechanics, stability, and regenerative performance for advanced GBR.

## Highlights

• TAWS strategy turns soft hydrogels into tough and oriented GBR barriers.
• TA crosslinking enables hydrogel to modulate osteogenic microenvironment.
• GHT membranes promote mandibular bone regeneration in inflammatory defects.

## Graphical abstract

**Figure:**
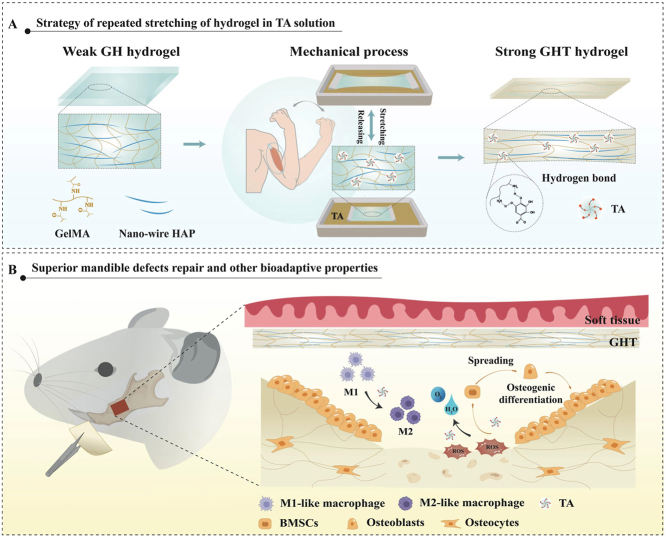

## 1 Introduction

Mandible defects resulting from trauma, surgical resection, inflammation and congenital malformation pose significant functional and psychological problems for patients, which often necessitate interventions such as guided bone regeneration (GBR) [[1], [2], [3]]. In GBR, barrier membranes play a central role by preventing soft tissue infiltration and maintaining the space necessary for osteogenic cells to populate the defect site and promote new bone formation [4]. Current GBR membranes are categorized into non-resorbable (e.g., ePTFE, titanium-reinforced membranes) and resorbable materials, most commonly collagen-based membranes. Although collagen-based resorbable membranes are generally preferred to avoid secondary surgical removal [5], most existing options lack sufficient mechanical robustness to withstand physiological stresses and adequate bioactivity to modulate the pathological microenvironment, both of which are critical determinants of regenerative success [[6], [7], [8]].

Beyond serving as passive physical barriers, GBR membranes are increasingly recognized as active regulators of the local biological microenvironment that critically shapes bone healing outcomes [9,10]. In the jawbone, persistent microbial colonization and inflammatory stimuli induce excessive production of reactive oxygen species (ROS), leading to sustained oxidative stress that disrupts normal regenerative processes. Elevated ROS levels impair osteoblast viability and activate redox-sensitive signaling pathways, such as NF-κB and MAPK, thereby suppressing osteogenic gene expression and matrix formation [11]. Concurrently, effective bone regeneration requires a timely phenotypic transition of macrophages from the pro-inflammatory M1 state to the pro-regenerative M2 state [[12], [13], [14]]. Prolonged M1 dominance maintains high levels of inflammatory cytokines, including TNF-α and IL-1β, promoting apoptosis of osteoblasts and mesenchymal stem cells while exacerbating osteoclast-mediated bone resorption [[15], [16], [17], [18], [19]]. In contrast, M2 macrophages facilitate resolution of inflammation by secreting IL-10 and support bone formation through the release of osteogenic factors such as TGF-β and BMP-2 [20,21]. Collectively, these insights highlight that successful GBR requires not only mechanical space maintenance but also active attenuation of oxidative stress and precise immunomodulation within the defect niche.

Recent GBR membranes have made significant progress in biofunctionalization aimed at regulating the local immune microenvironment. To this end, a wide range of bioactive components, including therapeutic metal ions, small-molecule drugs, and natural polyphenols, have been incorporated into collagen-based membranes to impart immunomodulatory and antioxidant functions [[22], [23], [24]]. For example, collagen membranes coated with nanoscale Ca_2_ZnSi_2_O_7_ bioactive glass release Ca^2+^, Zn^2+^, and silicate ions, thereby suppressing inflammation and enhancing osteoimmunomodulation [25]. Similarly, small-molecule drugs such as aspirin have been loaded into collagen/chitosan membranes to enable sustained local release, reducing inflammatory responses and promoting osteogenic differentiation [26,27]. In parallel, natural polyphenols, particularly epigallocatechin gallate (EGCG), are widely explored due to their strong redox activity; EGCG-modified collagen membranes suppress pro-inflammatory cytokine expression, promote CD206^+^ M2 macrophage polarization, and enhance the secretion of pro-regenerative factors [28,29]. Despite these advances, most collagen-based biofunctional membranes remain mechanically fragile and degrade rapidly under physiological conditions, increasing the risk of membrane collapse or soft-tissue invasion into the defect site and ultimately compromising bone augmentation [22,30,31]. Therefore, developing GBR membranes that integrate durable mechanical robustness with sustained immunoregulatory bioactivity remains highly desirable yet technically challenging.

Natural protein hydrogels, owing to their excellent biocompatibility and tunable degradability, have emerged as attractive candidates for next-generation GBR membranes [[32], [33], [34], [35]]. However, because of their disordered polymer networks and low effective crosslinking density, unmodified natural hydrogels are intrinsically mechanically weak and prone to excessive swelling, thereby compromising their space-maintaining function in GBR. To address this limitation, mechanical training has recently gained attention as a simple and scalable reinforcement strategy that avoids the introduction of additional chemical components. Inspired by the adaptive strengthening of tendons under repetitive mechanical loading, mechanically trained hydrogels are subjected to controlled deformation, such as uniaxial stretching in air [36,37], salt solutions [[38], [39], [40]], or other solutions [[41], [42], [43]], which induces polymer chain alignment and reduces intermolecular spacing, leading to enhanced mechanical strength [44]. These features make mechanical training appealing for fabricating mechanically reinforced hydrogel membranes for GBR. Nevertheless, most mechanically trained hydrogels rely predominantly on reversible chain alignment and possess limited intrinsic bioactivity. More critically, their reinforced structures tend to relax upon hydration and swelling under physiological conditions, resulting in rapid mechanical deterioration and insufficient long-term stability for GBR applications [45]. Together, these limitations highlight an unmet need for reinforcement strategies that can endow natural hydrogels with both durable mechanical integrity under physiological conditions and sustained biological functionality.

**Fig. 1:**
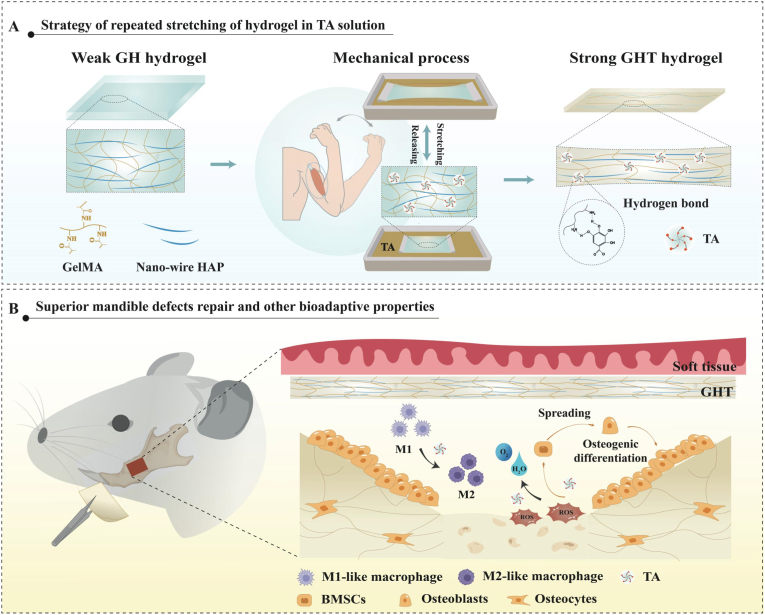
Schematic illustration of the TA-assisted wet-stretching (TAWS) strategy for fabricating oriented hydrogel barrier membranes. (A) Natural GelMA/HAp hydrogel undergoing TA-assisted mechanical training, inspired by tendon-strengthening mechanisms. (B) The TA-trained hydrogel regulates the osteogenic microenvironment of mandibular bone defects.

To address this gap and meet the multifaceted demands of guided bone regeneration, including long-term mechanical robustness, attenuation of oxidative stress, and immunomodulation—we propose a tendon-inspired tannic acid–assisted wet-stretching (TAWS) strategy for fabricating mechanically reinforced and intrinsically bioactive hydrogel membranes. In this design, the pristine hydrogel is first prepared by photopolymerization of GelMA, selected for its biocompatibility and enzymatic degradability [35,46,47], and reinforced with hydroxyapatite (HAp) nanowires to impart osteoconductivity [48,49]. Subsequent mechanical training in a tannic acid (TA) solution reorganizes and stabilizes the GelMA–HAp network, yielding a membrane with sustained mechanical integrity and biological functionality (Fig. 1). TA's polyphenolic backbone engages GelMA chains via multivalent hydrogen bonding with amide (–CONH–) and hydroxyl (–OH) groups, as well as π–π interactions with residual aromatic residues, forming reversible physical crosslinks that fix stretch-induced chain alignment and inhibit relaxation upon physiological swelling [[50], [51], [52], [53]]. Biologically, TA establishes a pro-regenerative microenvironment by directly scavenging ROS, including superoxide anions (O_2_^−^) and hydroxyl radicals (·OH) [54,55], and by enhancing endogenous antioxidant defenses through upregulation of superoxide dismutase and catalase and activation of Nrf2 signaling [56,57]. Using this platform, we systematically characterize the structural, mechanical, and biological properties of TAWS-engineered membranes, evaluate their capacity to support stem cell survival, proliferation, and osteogenic differentiation under oxidative stress in vitro, and assess their regenerative efficacy in a clinically relevant mandibular defect model in vivo. Collectively, this work demonstrates that integrating mechanically stabilized training with bioactive modulation yields a next-generation GBR membrane that reconciles durable structural support with active regulation of the regenerative microenvironment.

## 2 Materials and methods

### 2.1 Synthesis of GelMA and HAp-NWs

GelMA was synthesized as previously described [58]. 4 g of type A porcine skin gelatin was dissolved in 200 mL deionized water at 40 °C, and the pH was adjusted to 7.4 with sodium hydroxide. Dimethylformamide (60 mL) and methacrylic anhydride (300 μL) were then added, and the reaction was allowed to proceed at 40 °C for 2 h. The mixture was transferred into 1500 mL of anhydrous ethanol to induce precipitation. The resulting white fibrous product was collected, redissolved, and freeze-dried to yield GelMA. The degree of substitution (DS) of GelMA was analyzed in deuterium oxide (D_2_O) at a frequency of 400 MHz (AVANCE III HD400, Bruker, Germany). The spectra were normalized to the phenylalanine signal (6.9–7.5 ppm). Then the integrated intensities of lysine methylene at 2.8–2.95 ppm in the spectra of gelatin and GelMA were measured, respectively. The DS of GelMA was determined by the formula as follows:

$$DS=\frac{\int \text{Gelatin}\;\left(2.8-2.95\;\text{ppm}\right)-\int \text{GelMA}\left(2.8-2.95\;\text{ppm}\right)}{\int \text{Gelatin}\;\left(2.8-2.95\;\text{ppm}\right)}$$

HAp-NWs were prepared via a hydrothermal method. CaCl_2_ (0.735 g), NaOH (2.5 g), and NaH_2_PO_4_ (0.6 g) were each dissolved in 50 mL, 40 mL, and 20 mL deionized water, respectively. Oleic acid (40 mL) and anhydrous ethanol (30 mL) were mixed and stirred magnetically for 10 min. The prepared CaCl_2_, NaOH, and NaH_2_PO_4_ solutions were then added dropwise to the oleic acid–ethanol mixture under vigorous stirring. After 20–30 min of reaction at room temperature, the solution was transferred to a 100 mL Teflon-lined stainless steel autoclave and heated at 180 °C for 12 h. Following natural cooling, the precipitate was collected, washed thoroughly with anhydrous ethanol, and redispersed in ethanol for storage. The phase composition of HAp-NWs were analyzed by X-ray diffraction (XRD; Ultima IV).

### 2.2 Mechanical training process

One end of the pristine hydrogel was fixed to the clamp of a universal testing machine, while the other end was secured to a clamp inside a custom-made uncovered box-shaped container (Fig. 1A). Given that the initial hydrogel was soft and prone to breakage, soft and rough cotton tape was used to fix the hydrogel at both ends, ensuring that no sharp clamping-induced damage would affect the results. A tannic acid (TA) solution of a specific concentration was poured into the custom container, with the liquid level maintained to ensure the hydrogel remained fully submerged during the stretching process. The training involved cyclic stretching-relaxation with a target displacement of 120 % and consistent hold times (30 s at maximum stretch and 30 s at original site), while adjusting training durations to match three preset speeds (20, 40, and 80 mm min^−1^). The number of cycles was adjusted based on individual cycle duration to ensure uniform total mechanical stimulation. Immediately after the stretching process, the hydrogel was immersed in PBS and rinsed repeatedly to remove free TA molecules (Table 1.).

**Table 1:** Cyclic stretching-relaxation parameters for mechanical training.

Stretching Speed	Stretching Phase Duration	Relaxation phase Duration	Hold Time at Max Stretch and Original site	Total Cycle Duration
20 mm min^−1^	90 s	90 s	30 s for each	4 min
40 mm min^−1^	45 s	45 s	30 s for each	2.5 min
80 mm min^−1^	22.5 s	22.5 s	30 s for each	105 s

### 2.3 Fabrication of G, GH, GHS, and GHT hydrogels

GelMA was dissolved in deionized water (10 % w/v) with lithium phenyl-2,4,6-trimethylbenzoylphosphinate (LAP) (0.2 % w/v) and photocrosslinked under visible light (6.9 mW cm^−2^, 400–480 nm, 5 min) to form GelMA (G) hydrogels. For GelMA–HAp (GH) hydrogels, HAp-NWs (1–1.5 % w/v) were added to the same precursor and photocrosslinked under identical conditions. GelMA-HAp-TA soaked (GHS) hydrogels were obtained by soaking GH in tannic acid (TA) solution (2, 5, 10, 20, 30 % w/v) for 24 h, followed by PBS rinsing to remove unbound TA. For GelMA-HAp-TA trained (GHT) hydrogels, GH samples were immersed in TA solution (2–30 % w/v) and subjected to cyclic stretching (0.5–4 h) using a universal testing machine, then rinsed in PBS to remove residual TA. Sample abbreviations are defined in Table S3 for clarity.

### 2.4 Characterization of GHT hydrogels

#### 2.4.1 Physicochemical characterization

The microstructures of hydrogels were examined by scanning electron microscopy (SEM). Freeze-dried samples were imaged using a Hitachi S-4800 (15 kV). The surface elemental composition and chemical states of the hydrogels were characterized by X-ray photoelectron spectroscopy (XPS; Thermo Kalpha). Raman spectroscopy (Thermo Fisher DXR) was employed to further analyze molecular vibrations and structural features of the hydrogels. Molecular structure and bond interactions were analyzed using Fourier transform infrared spectroscopy (FTIR; Nicolet iS10, Thermo). Small-angle X-ray scattering (SAXS; XEUSS 2.0, Xenocs) was used to assess structural orientation. SAXS was performed at λ = 1.54 Å with a sample-to-detector distance of 1188 mm, calibrated with silver behenate. All spectra were background-subtracted.

#### 2.4.2 Mechanical testing

Uniaxial tensile tests were performed on an Instron 5944 (50 N load cell). Hydrogels were swollen in PBS for 24 h and cut into strips (50 mm × 8 mm), and the samples were stretched at 5 mm min^−1^. Stress–strain curves were recorded, where elastic modulus was calculated from the 5–15 % strain region and toughness from the area under the curve.

#### 2.4.3 Swelling and degradation properties of membranes

For swelling tests, samples were weighed dry (Wd), immersed in PBS at 37 °C, and reweighed (Ws) after surface blotting at set timepoints (15 min, 30 min, 1 h, 2 h, 4 h, 10 h, 24 h). Swelling ratio was calculated as:

$$\text{Swelling}\;\text{ratio}\;\left(\%\right)=\frac{Ws-Wd}{Wd}\times 100$$

For degradation, freeze-dried samples were weighed (W_0_), placed in simulated body fluid (SBF, 37 °C), and reweighed after freeze-drying at preset intervals (Wt) (1 d, 3 d, 7 d, 14 d, 21 d, 32 d). Weight loss was calculated as:

$$\text{Weight}\;\text{loss}\;\left(\%\right)=\frac{W_{0}-Wt}{W_{0}}\times 100$$

#### 2.4.4 In vitro TA release kinetics assay

TA release was quantified by UV–Vis spectrophotometry based on its characteristic absorption at 276 nm. A calibration curve was established using TA standard solutions (1–100 μg mL^−1^). Hydrogel samples of equal volume were immersed in 10 mL of artificial saliva and incubated at 37 °C under constant shaking. At predetermined time points, 500 μL of the release medium was collected for TA quantification by measuring absorbance at 276 nm and immediately replenished with an equal volume of prewarmed artificial saliva to maintain a constant volume.

### 2.5 In-vitro bioactivity of GHT hydrogels

#### 2.5.1 Cell culture

All procedures were approved by the Ethics Committee of the School of Stomatology, Shandong University (Approval No. 20220027) and conformed to NIH animal care guidelines. Rat bone marrow stromal cells (BMSCs) were isolated from male Sprague-Dawley rats (80–100 g). After euthanasia, hindlimb bones were harvested, disinfected, and flushed with α-MEM. Cells were cultured in α-MEM with 20 % FBS and 1 % penicillin–streptomycin, and passages 3–6 were used. L929 fibroblasts and RAW264.7 macrophages (Cell Bank, Chinese Academy of Sciences) were cultured in α-MEM or DMEM with 10 % FBS at 37 °C and 5 % CO_2_ [59].

#### 2.5.2 Cell viability assay and live/dead staining

For the extract preparation, membrane specimens (10 mm × 10 mm × 0.5 mm) were immersed in complete culture medium at a ratio of 0.2 g mL^−1^ and incubated for 24 h at 37 °C, following ISO 10993-12 standards. All exposed surfaces, including the side faces, were fully immersed during extraction to ensure complete exposure.

BMSCs (5 × 10^3^ cells/well) were seeded in 96-well plates. BMSCs cultured in complete α-MEM without H_2_O_2_ or hydrogel extracts served as the negative control (Ctrl group), whereas cells treated with 400 μM H_2_O_2_ alone served as the positive control (H_2_O_2_ group). Cells were pretreated with hydrogel extracts from each group for 24 h, and were then treated with or without H_2_O_2_ for 2 h. CCK-8 reagent (10 μL) was added after treatment and absorbance at 450 nm was recorded after 2 h. Cell viability was expressed relative to untreated controls. For Live/Dead staining, cells were cultured with extracts for 48 h, then stained with Calcein-AM (2 μM) and propidium iodide (4.5 μM), and imaged under a fluorescence microscope.

#### 2.5.3 Barrier function experiments

GHT hydrogels after being soaked in alcohol and disinfected with ultraviolet light were placed in transwell inserts, and 5 × 10^4^ L929 fibroblasts (a model soft tissue cell line) were seeded on the surface of each hydrogel. Hydrogel membranes in the upper chambers were immersed in α-MEM containing 1 % FBS, while the lower chambers contained α-MEM with 5 % FBS to generate chemotactic cues. Following 72 h of incubation, hydrogels were removed, and cells on both surfaces were stained with DAPI.

#### 2.5.4 Migration experiments

BMSCs migration was evaluated using 24-well Transwell inserts with 8 μm pore polycarbonate membranes. Briefly, 5 × 10^4^ cells were seeded into the upper chambers in α-MEM containing 1 % FBS, while the lower chambers contained hydrogel membranes immersed in α-MEM with 5 % FBS to generate chemotactic cues. After 24 h of incubation, non-migrated cells on the upper membrane surface were gently removed with a cotton swab. Migrated cells on the underside were fixed with 4 % paraformaldehyde, stained with 0.1 % crystal violet, imaged using a light microscope (KEYENCE, Osaka, Japan), and quantified by counting five random fields per sample. Cell migration was also evaluated using a scratch wound healing assay. BMSCs (2 × 10^5^ cells/well) were seeded in 6-well plates and grown to confluence. A linear scratch was created with a pipette tip, washed with PBS, and incubated with hydrogel extracts. Images were collected at 0, 24, and 48 h. Wound closure was quantified with ImageJ.

#### 2.5.5 Flow cytometry analysis

Intracellular ROS levels in BMSCs were quantified using DCFH-DA. Untreated BMSCs (no H_2_O_2_ or hydrogel extracts) served as the negative control (Ctrl group), whereas BMSCs treated with 400 μM H_2_O_2_ alone served as the positive control (H_2_O_2_ group). After being cultured with extracts from each group for 24 h, cells were treated with or without 400 μM H_2_O_2_ for 2 h. After that, cells were digested and then incubated with 10 μM DCFH-DA at 37 °C for 30 min, washed three times with PBS, and analyzed on a flow cytometer (BD Biosciences, USA). Fluorescence intensity was used to assess ROS accumulation. For RAW264.7 macrophages, polarization markers were evaluated by staining with fluorescent antibodies. After treatment, cells were washed with PBS and incubated with anti-CD86 (BioLegend, 159204) for 30 min at 4 °C in the dark. Cells were then permeabilized and stained with anti-CD206 (BioLegend, 141708) for 30 min under light-protected conditions. After washing, cells were resuspended in PBS and analyzed by flow cytometry. The proportions of CD86^+^ (M1) and CD206^+^ (M2) populations were quantified.

#### 2.5.6 Free radical scavenging assays

DPPH scavenging activity was assessed by mixing each sample (100 μL) with ethanol-based DPPH solution (700 μL, 0.1 mM). DPPH solution mixed with PBS instead of hydrogel extracts was the negative control (Ctrl group), and 100 μM ascorbic acid was the positive control. After incubation in the dark for 30 min at room temperature, absorbance was recorded at 512 nm using a microplate reader (BioTek, USA). Scavenging efficiency was calculated as:

$$\text{Scavenging}\;\left(\%\right)=\frac{A_{0}-A_{1}}{A_{0}}\times 100$$

where $A_{0}$ is the absorbance of the negative control (DPPH + PBS) and $A_{1}$ is that of the sample-treated solution.

Copper ion-mediated Fenton-like reaction can convert H_2_O_2_ into ⋅OH, which can be assessed by 3,3′,5,5′-Tetramethylbenzidine (TMB). Briefly, 800 μL deionized water, 20 μL H_2_O_2_ (3 %), 10 μL TMB (20 mg mL^−1^) and 20 μL CuCl_2_ (100 μg mL^−1^) were mixed with 20 μL sample extract. PBS was used as the negative control, and ascorbic acid was used as the positive control. After being incubated for 15 min at room temperature, absorbance was measured at 652 nm. Scavenging percentage was calculated using the same equation.

Superoxide scavenging was quantified using an O_2_^−^ Activity Assay Kit (Solarbio, China) according to the manufacturer's protocol, with PBS as the negative control. Absorbance was recorded at 530 nm, and scavenging efficiency was calculated as above.

#### 2.5.7 Intracellular ROS detection

Intracellular ROS levels were assessed using a Reactive Oxygen Species Assay Kit (Beyotime, China). BMSCs were seeded in 24-well plates at 2.5 × 10^4^ cells per well and allowed to adhere. Cells were incubated with hydrogel extract solutions for 24 h and then exposed to 400 μM H_2_O_2_ in α-MEM for 2 h. After treatment, cells were stained with 10 μM DCFH-DA for 30 min at 37 °C, washed three times with PBS, and imaged using a fluorescence microscope (KEYENCE, Japan). To assess redox homeostasis, the GSH/GSSG ratio was measured in BMSCs treated with H_2_O_2_ and hydrogel extracts using a commercial assay kit (Beyotime, China).

#### 2.5.8 Mitochondrial function analysis

Mitochondrial activity and oxidative stress were assessed using MitoTracker Deep Red and MitoSOX (Beyotime, China), respectively. For mitochondrial activity, cells were incubated with hydrogel extract solutions for 24 h and then exposed to 400 μM H_2_O_2_ in α-MEM for 2 h, followed by staining with MitoTracker Deep Red for 30 min at 37 °C and Hoechst 33342 for 5 min. Samples were imaged using a confocal laser scanning microscope (LSM980, ZEISS).

For mtROS detection, after treatment, cells were incubated with the MitoSOX probe for 30 min at 37 °C, stained with Hoechst 33342, and imaged using a fluorescence microscope (KEYENCE, Japan).

#### 2.5.9 Osteogenic induction of BMSCs

BMSCs at passages 3–4 were seeded in 6- or 12-well plates and induced once cultures reached 70–80 % confluence. Osteogenic medium consisted of α-MEM with 10 % FBS, 50 mg L^−1^ ascorbic acid, 10 mM β-glycerophosphate, and 10^−8^ M dexamethasone. The medium was refreshed every 3 days throughout the induction period.

#### 2.5.10 Alkaline phosphatase (ALP) staining and Alizarin Red S (ARS)

To evaluate osteogenic differentiation, BMSCs were seeded in 6-well plates (1 × 10^5^ cells/well) and stimulated with 400 μM H_2_O_2_ for 2 h every 2 days within the initial 5 days. Meanwhile, the cells were cultured in osteogenic medium containing hydrogel extracts. On day 7, cells were fixed with 4 % paraformaldehyde and stained for ALP using a BCIP/NBT kit (Beyotime, China). On day 21, Alizarin Red S staining was performed to assess matrix mineralization. Staining was visualized using a fluorescence microscope (KEYENCE, Japan). For quantification, ALP activity was measured using an alkaline phosphatase assay kit (Beyotime, China). For ARS, calcium deposits were solubilized in 10 % cetylpyridinium chloride, and absorbance was recorded.

#### 2.5.11 Quantitative real-time PCR (qRT-PCR) and enzyme-linked immunosorbent assay (ELISA)

For antioxidant analysis, BMSCs were first treated with extracts for 24 h, and then stimulated with 400 μM H_2_O_2_ for 2 h, and expression of SOD-1 and CAT was quantified by qRT-PCR. For ALP and OPN expression, cells were stimulated with 400 μM H_2_O_2_ for 2 h every 2 days within the initial 5 days, and simultaneously induced for 7 days before assessed. For immunomodulation studies, RAW264.7 macrophages were stimulated with lipopolysaccharide (LPS) (200 ng mL^−1^) for 24 h, followed by treatment with extracts for 6 h, and then mRNA levels of TNF-α, iNOS, Arg-1, and IL-10 were measured.

Total RNA was extracted using TRIzol (Invitrogen, USA), reverse-transcribed with the PrimeScript RT Kit (Takara, China), and amplified using SYBR Premix Ex Taq on a Bio-Rad CFX96 system. Gene expression was calculated using the 2^−^ΔΔCt method. Primer sequences are listed in Tables S1 and S2.

Supernatants from RAW264.7 cultures were collected, and TNF-α and IL-10 levels were quantified by ELISA (Lianke, China).

#### 2.5.12 Immunofluorescence staining

Immunofluorescence was used to assess osteogenic differentiation in BMSCs and macrophage polarization in RAW264.7 cells. After the same treatment as mentioned above, cells were fixed with 4 % paraformaldehyde for 15 min, permeabilized with 0.1 % Triton X-100 for 10 min, and blocked with 5 % BSA for 1 h. For BMSCs, cells were incubated overnight at 4 °C with primary antibodies against RUNX2 (1:500, Proteintech) and OPN (1:500, Proteintech). After PBS washing, appropriate CoraLite594- or CoraLite488-conjugated secondary antibodies (1:1000, Proteintech) were applied for 1 h at room temperature in the dark, followed by nuclear staining with DAPI (Servicebio, China). RAW264.7 cells underwent the same fixation, permeabilization, and blocking steps, then were stained with primary antibodies against CD86 (1:500) and CD206 (1:500), followed by fluorescent secondary antibodies and DAPI counterstaining. Images were acquired on a confocal laser scanning microscope (LSM980, ZEISS), and fluorescence intensity was quantified using ImageJ.

### 2.6 In-vivo bone regeneration of GHT hydrogels

#### 2.6.1 Rat mandibular bone defect model

All animal procedures were approved by the Medical Ethics Committee of the School of Stomatology, Shandong University (Protocol ID: 20220027) and conducted in accordance with the NIH Guide for the Care and Use of Laboratory Animals. Wistar rats (8 weeks, 200–250 g, male, SPF) were used to establish a mandibular bone defect model. The 60 rats were randomly divided into five groups using a computer-generated random number table: ROSup, ROSup + G, ROSup + GH, ROSup + GHT and ROSup + Bio-Gide® groups, each with 12 rats. Animals were euthanized at 1 week (4 rats per group) for ROS and inflammation analysis, 4 weeks (4 rats per group) and 8 weeks (4 rats per group) for bone regeneration analysis. Rats were anesthetized with 1 % sodium pentobarbital (40 mg kg^−1^), with additional dosing as needed. After shaving and aseptic preparation, a 10 mm incision was made parallel to the mandibular ramus, ∼2 mm above the inferior border. Soft tissues were gently separated to expose the buccal cortical bone. A standardized defect (5 × 4 × 1 mm^3^) was created using a high-speed fissure burr, located 1 mm posterior to the anterior margin and 1 mm inferior to the superior edge of the mandible (Fig. S12). To induce an ROS-enhanced microenvironment, 1 mg mL^−1^ LPS (Sigma-Aldrich) in PBS was applied to the defect. Each membrane was cut into a 6 × 5 mm^2^ sheet with a thickness of 1 mm to ensure full coverage of the defect, and the incision was closed in layers. Postoperatively, rats received intramuscular penicillin (80,000 IU per day) for 3 days. Animals were euthanized at the first week for the analysis of ROS levels and inflammatory status, while those at the 4 and 8 weeks were euthanized to evaluate osteogenic outcomes.

#### 2.6.2 In vivo ROS detection

Fresh, unfixed mandibular specimens were cryosectioned at 10–20 μm thickness and incubated with dihydroethidium (Bestbio, China) for 30 min. Sections were then imaged using a confocal laser scanning microscope (LSM980, ZEISS) to assess ROS distribution.

#### 2.6.3 Micro-CT analysis

Mandibular specimens were fixed in 10 % formalin and scanned using a Quantum GX micro-CT system (PerkinElmer, USA) at 90 kV, 88 μA, and 50 μm voxel resolution. 3D reconstruction was performed using the system software, and quantitative analysis was conducted in MATLAB (R2018b) and 3D Slicer (v4.8.1). ROI was strictly confined to the entire mandibular bone defect area (5 × 4 × 1 mm^3^) and the surrounding 0.5 mm of native bone tissue to avoid interference from irrelevant structures. Bone volume fraction (BV/TV) and bone mineral density (BMD) were evaluated as key indicators of regeneration.

#### 2.6.4 H&E, Masson, TRAP staining

Mandibular specimens were fixed in 4 % paraformaldehyde for 24–48 h, decalcified in 10 % EDTA for 4 weeks, embedded in paraffin, and sectioned at 4 μm thickness. Hematoxylin–eosin (H&E; Solarbio) staining was performed to assess bone morphology and defect healing. Collagen deposition and fibrotic tissue were evaluated using Masson's trichrome staining (Solarbio). Osteoclast activity was assessed by tartrate-resistant acid phosphatase (TRAP) staining (Solarbio) with hematoxylin counterstaining. H&E, Masson and TRAP were imaged using bright-field microscopy (LSM980, ZEISS).

#### 2.6.5 Immunohistochemistry and immunofluorescence

Paraffin sections were dewaxed, rehydrated, and subjected to antigen retrieval in citrate buffer. For immunohistochemistry (IHC), sections were blocked with 3 % H_2_O_2_ and 5 % BSA, then incubated with primary antibodies against RUNX2 and OCN (1:500, Proteintech). HRP-conjugated secondary antibodies (1:4000, Proteintech) were applied and visualized using DAB. For immunofluorescence (IF), sections were incubated with primary antibodies against TNF-α (1:300), CD206 (1:10,000), and OPN (1:500), followed by fluorescent secondary antibodies (goat anti-rabbit 488, 1:200; goat anti-mouse 594, 1:500). Nuclei were counterstained with DAPI. IHC samples were imaged using bright-field microscopy, while IF sections were visualized by fluorescence microscopy (LSM980, ZEISS). Quantitative analysis was performed using ImageJ.

### 2.7 Statistical analysis

All data were presented as the mean ± standard deviation (S.D.) from a minimum of three independent experiments. Statistical comparisons were performed by Student's *t*-tests or one-way analysis of variance (ANOVA). The values with *p* < 0.05 were considered significant (represented as ∗*p* < 0.05, ∗∗*p* < 0.01, ∗∗∗*p* < 0.001, or not significant (ns)). ImageJ (version 1.8.0) was utilized for in vitro and in vivo imaging analysis. Data were analyzed using GraphPad Prism 10.0 software (GraphPad Prism, San Diego, California, USA).

## 3 Results

### 3.1 Design and characterization of GHT hydrogel membranes

**Fig. 2:**
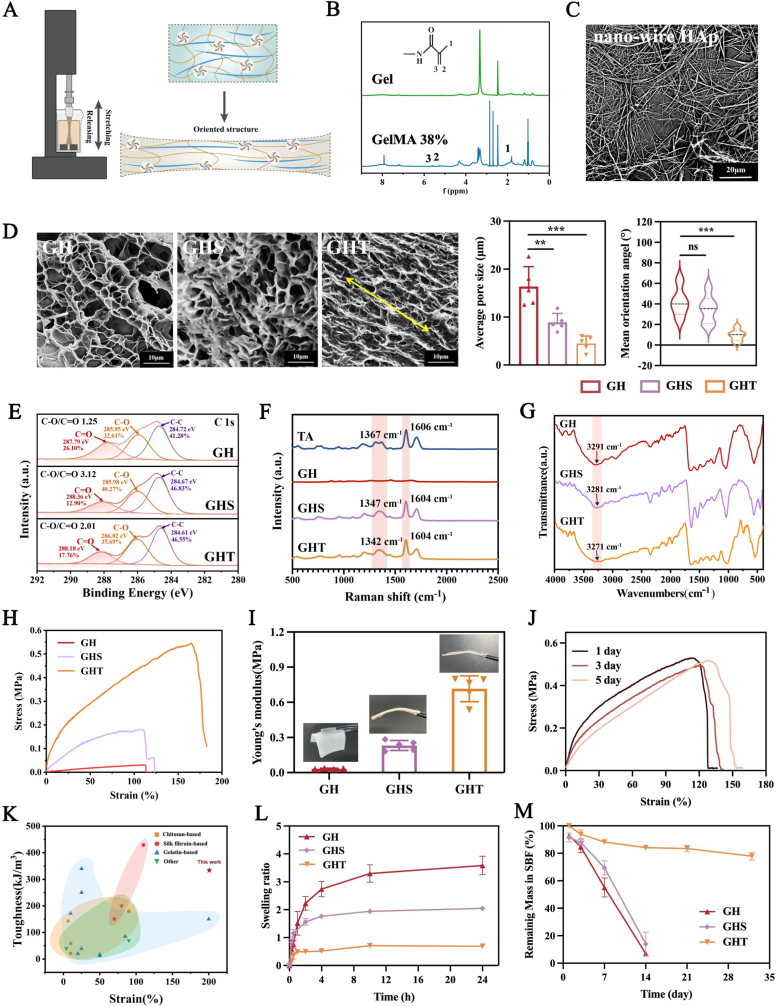
Characterization of hydrogels. (A) Schematic diagram of dynamic stretching of hydrogel in TA solution. (B) ^1^H NMR spectra of GelMA. (C) SEM images of HAp nanowires (HAp-NWs). (D) SEM images and quantitative analysis of pore size and orientation angle of the freeze-dried GH, GHS and GHT. (E) High-resolution XPS spectra of C 1s, (F) Raman spectra and (G) FTIR spectra of GH, GHS and GHT. (H) Stress-strain curves, (I) Young's modulus and digital photos of GH, GHS and GHT. (J) Stress-strain curves of GH, GHS and GHT at 1, 3, and 5 days post-swelling in artificial saliva. (K) Comparative analysis of GHT hydrogels and previously reported natural hydrogel-based GBR membranes in terms of tensile strain and toughness. Chitosan-based [[64], [65], [66], [67]]; Silk fibroin-based [46,68]; GelMA-based [46,[69], [70], [71], [72], [73], [74], [75]]; others (e.g., Alginate-based, Collagen-based) [[76], [77], [78]]. (L) Swelling curves and (M) remaining mass of GH, GHS, GHT in SBF. (n = 5 for each group) Data are means ± SD; ns: No significant differences, ∗∗*p* < 0.01, ∗∗∗*p* < 0.001.

The bioinspired tannic acid (TA)-assisted wet-stretching (TAWS) strategy for fabricating multifunctional GBR membranes comprises two sequential steps. First, GelMA was mixed with hydroxyapatite nanowires (HAp-NWs) and photopolymerized to form a GelMA-HAp (GH) composite hydrogel, in which HAp-NWs enhance both the mechanical flexibility and osteogenic potential of the hydrogel. Here, one-dimensional HAp nanowires were chosen as reinforcing fillers due to their high aspect ratio, which enables more efficient reinforcement of GelMA than spherical nanoparticles by forming percolated networks that facilitate stress transfer and crack bridging at low loadings [[60], [61], [62], [63]]. Subsequently, the GH hydrogel undergoes mechanical training (repeated cycles of stretching and relaxation) in a TA solution (Fig. 2A). This process induces molecular chain alignment along the loading axis while simultaneously promoting multivalent hydrogen bonding between GelMA and TA. The resulting interactions immobilize the oriented polymer network, yielding a strong, tough, and swelling-resistant GelMA-HAp-TA (GHT) hydrogel.

Successful synthesis of GelMA with a degree of substitution (DS) of 38 % was confirmed by ^1^H NMR spectroscopy (Fig. 2B). HAp-NWs were synthesized via a hydrothermal method using calcium oleate as a precursor. SEM confirmed the formation of uniform nanowires with lengths of 50–200 μm and widths of 30–50 nm (Fig. 2C), while XRD analysis verified the phase purity of HAp-NWs, showing characteristic hydroxyapatite peaks (JCPDS No. 09–0432) without impurities (Fig. S1). These HAp-NWs were then incorporated into GelMA at varying mass ratios (GelMA:HAp = 10:1, 10:1.5) to fabricate GH composite hydrogels. SEM imaging revealed a loose, porous microstructure within the GH composites (Fig. S2A), and Energy-dispersive X-ray spectroscopy (EDS) mapping further confirmed the non-uniform distribution of constituent elements (C, O, N, Ca, P) throughout the hydrogel matrix (Fig. S2B). Critically, HAp incorporation significantly enhanced the mechanical properties of the hydrogel, increasing both strength and flexibility (Fig. S2C and D), thereby facilitating the subsequent tensile training process. The composite with 1.5 % HAp-NWs (GelMA:HAp = 10:1.5) demonstrated optimal mechanical performance, exhibiting a 4.67-fold increase in Young's modulus and a 7.86-fold enhancement in tensile strength compared to pure GelMA (G). This composition was therefore selected for subsequent mechanical training.

To evaluate the influence of mechanical training on hydrogel pore structure alignment, the microstructures of the pristine GH hydrogel, the GH hydrogel soaked in tannic acid (TA) solution (GHS), and the GH hydrogel subjected to mechanical training (GHT) were characterized using SEM. As shown in Fig. 2D, the pristine GH hydrogel exhibited a typical isotropic honeycomb porous structure. The GHS hydrogel was characterized by a dense pore structure (7–12 μm) with reduced pore diameters and thickened pore walls due to the increased crosslinking density, while GHT hydrogel displayed a significantly denser microstructure (3–6 μm) featuring oriented pore walls aligned parallel to the stretching direction. Pore orientation analysis revealed that the internal pore structure of GHT became elliptical, with its long axis aligned within 20° of the tensile direction. In contrast, both GH and GHS hydrogels exhibited nearly isotropic pore distributions, with orientation angles averaging approximately 40°. This observation indicates that mechanical stretching within the TA medium not only induces directional alignment of the hydrogel microstructure but also enhances bonding interactions between GelMA and TA.

XPS analysis confirmed the successful incorporation of TA into both GHS and GHT hydrogels (Fig. S3 and 2E). In the C 1s spectra, the C–O/C=O ratio increased from 1.25 in GH to 3.12 in GHS and 2.01 in GHT, reflecting the introduction of abundant hydroxyl groups from TA. Raman spectroscopy further revealed GelMA–TA interactions in GHS and GHT (Fig. 2F), as evidenced by a red shift of the phenolic C–O stretching peak from 1367 cm^−1^ to 1347 cm^−1^ in GHS and to 1342 cm^−1^ in GHT. Consistently, FTIR spectra showed a red shift of the O–H stretching band from 3291 cm^−1^ in GH to 3281 cm^−1^ in GHS and 3271 cm^−1^ in GHT, indicating progressively strengthened hydrogen bonding (Fig. 2G). Notably, compared with simple soaking (GHS hydrogel), mechanical training (GHT hydrogel) further enhanced GelMA–TA interactions. Collectively, these spectral changes demonstrate that in GHT hydrogels, TA forms stable hydrogen-bond-mediated interactions that “lock” the oriented polymer network.

Next, the stress-strain curves of GH, GHS, and GHT hydrogels demonstrated that mechanical training within the TA solution transformed the brittle GH hydrogel into a tough GHT hydrogel while maintaining high tensile strain, with significantly improved toughness observed in both macroscopic behavior and tensile testing (Fig. 2H–S4). Quantitatively, the GHT hydrogel exhibited a 22.16-fold increase in Young's modulus (from 0.032 MPa to 0.716 MPa) compared to the pristine GH hydrogel (Fig. 2I). This pronounced enhancement is attributed to the synergistic effects of polymer chain alignment and strong GelMA–TA interactions, which collectively convert a disordered, loosely crosslinked network into a structurally robust one. Notably, the TAWS strategy reinforced the hydrogel not only along the tensile direction but also in perpendicular directions (Fig. S5). The long-term mechanical stability of GHT membranes was further evaluated by immersion in artificial saliva for 5 days. GHT hydrogels exhibited only minimal strength changes over this period (Fig. 2J), in sharp contrast to hydrogels mechanically trained in saline solution (Fig. S6). This stability confirms that GHT membranes retain structural integrity and load-bearing capacity in a simulated oral microenvironment, supporting their suitability for GBR applications. Moreover, comparison with reported natural hydrogel-based GBR membranes highlights the superior toughness and flexibility achieved by the TAWS strategy (Fig. 2K).

Natural hydrogels typically undergo substantial swelling under physiological conditions, resulting in rapid deterioration of mechanical properties that critically compromise their barrier function. As shown in Fig. 2L, untreated GH hydrogels absorbed water rapidly, exhibiting a swelling ratio close to 400 %, while soaking in TA reduced the swelling ratio of GHS membranes to 200 %. Notably, GHT membranes demonstrated significantly enhanced stability with a swelling ratio of only 100 %. Degradation studies assessed these samples’ mass loss in simulated body fluid (SBF) at 37 °C over time (Fig. 2M), showing that GH hydrogel membranes degraded almost completely within 14 days, GHS membranes retained <20 % of their initial mass after the same period, while GHT membranes maintained about 80 % of their original mass after 28 days. Collectively, these results demonstrate that the proposed TAWS strategy endows the GHT hydrogel with high mechanical strength, swelling resistance, and appropriate degradation kinetics.

### 3.2 Effect of processing parameters on the performance of GHT membranes

Having established the principle of the TAWS strategy, we next optimized key process parameters, such as cyclic training duration, stretching rate, and tannic acid (TA) concentration, to maximize mechanical performance while maintaining cytocompatibility.

**Fig. 3:**
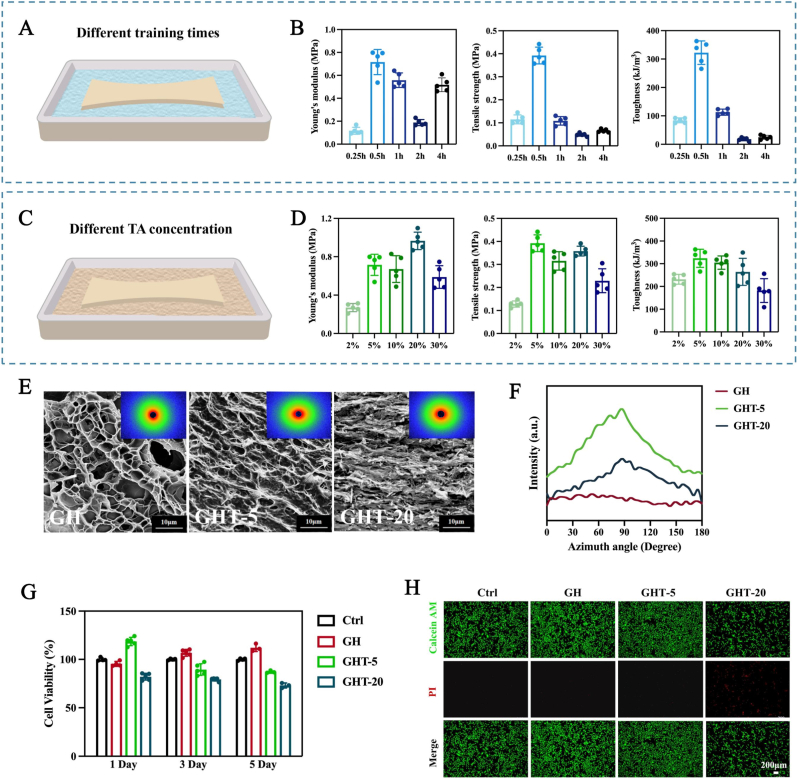
Optimization of TAWS processing parameters and their effects on GHT hydrogel performance. (A–B) Young's modulus, tensile strength, and toughness of GHT hydrogels as a function of training duration. (C–D) Mechanical properties of GHT hydrogels at different tannic acid (TA) concentrations. (E) SEM images and corresponding 2D SAXS patterns of GH, GHT-5, and GHT-20 hydrogels. (F) Azimuthally integrated SAXS intensity profiles showing changes in anisotropy. (G) The viability of cells cultured with hydrogel extracts for 1, 3, and 5 days. (H) Live/dead staining of cells after 48 h exposure to hydrogel extracts (Calcein-AM for live cells, green; PI (propidium iodide) for dead cells, red). (n = 5 for each group) Data are means ± SD.

The optimization of TAWS parameters was conducted through a stepwise manner, considering the coupled effects of mechanical loading kinetics and TA-polymer interactions. To decouple these variables, we first fixed the TA concentration at 30 % (w/v) and optimized the cyclic training duration and stretching rate. At a constant stretching speed of 20 mm min^−1^, training duration was varied from 0.25 to 4 h (Fig. 3A). Increasing the duration from 0.25 to 0.5 h resulted in a pronounced enhancement of mechanical performance, with Young's modulus and toughness increasing by 6.11-fold and 3.15-fold, respectively, relative to the 0.25 h condition (Fig. 3B). However, further prolonging the training duration led to a marked deterioration in mechanical properties; specifically, at 2 h the modulus decreased to approximately 25 % of the 0.5 h value. This reduction is attributed to plasticization and network over-relaxation caused by prolonged exposure to high TA content, which disrupts effective load-bearing chain alignment. Accordingly, 0.5 h was identified as the optimal training duration.

Using this optimized duration (0.5 h), we next evaluated the influence of stretching rate (20, 40, and 80 mm min^−1^). Increasing the stretching rate significantly weakened mechanical reinforcement, with rates ≥40 mm min^−1^ causing a pronounced decline in both modulus and toughness (Fig. S7). This behavior indicates that lower stretching rates allow the hydrogel to remain stretched for a longer period during each cycle, facilitating polymer chain alignment and TA-mediated hydrogen-bond locking. Therefore, a stretching speed of 20 mm min^−1^ was selected.

Under these optimized stretching conditions (20 mm min^−1^ for 0.5 h), we systematically investigated the effect of TA concentration (2, 5, 10, 20, and 30 % w/v) on the mechanical properties of GHT membranes (Fig. 3C). Samples were denoted as GHT-X, where X represents the TA concentration. Compared with pristine GH hydrogels, GHT-5 exhibited the most pronounced mechanical enhancement. Increasing TA concentration from 2 % to 5 % led to substantial increases in Young's modulus and tensile strength; however, further increases beyond 5 % resulted in diminishing or even negative returns. Notably, GHT-30 retained only ∼79 % of the Young's modulus of GHT-5, while tensile strength and toughness dropped to ∼50 % of the GHT-5 values (Fig. 3D). This trend likely reflects saturation of available hydrogen-bonding sites within the GelMA network, beyond which excess TA no longer contributes to effective load transfer and instead induces network plasticization. Notably, in the absence of mechanical training, TA treatment also increased the Young's modulus and tensile strength of GHS hydrogels in a concentration-dependent manner; however, these values remained far inferior to those of the GHT hydrogels (Fig. S8).

The microstructures of GHT hydrogels prepared with different TA concentrations were examined by SEM and small-angle X-ray scattering (SAXS) (Fig. 3E). SEM images and SAXS patterns of pristine GH hydrogels revealed isotropic structures with uniform scattering halos. After TAWS processing, however, elliptical scattering patterns were observed. The gradual sharpening of azimuthal intensity at 90° confirmed that mechanical training progressively increased hydrogel anisotropy (Fig. 3F).

To evaluate the biocompatibility of hydrogels mechanically trained at different tannic acid (TA) concentrations, cell viability was assessed using CCK-8 assays and Live/Dead staining. The untrained GH hydrogel exhibited excellent cytocompatibility, with cell viability consistently exceeding 100 %. In contrast, mechanically trained hydrogels showed a gradual decrease in viability with increasing TA concentration. This trend is expected, as excessive TA exposure can induce extrinsic apoptosis through the formation of phenol–protein or phenol–lipid complexes in the presence of oxygen (Fig. 3G). Accordingly, a low TA concentration (5 %) was selected as the optimal training condition, as the GHT-5 hydrogel exhibited no significant cytotoxicity and maintained over 80 % cell viability after 5 days. Live/Dead staining after 48 h of culture further confirmed the cytocompatibility of GHT-5 (Fig. 3H).

Notably, TA release profiles from GHS and GHT hydrogels exhibited distinct kinetics. GHS-5 displayed a burst release, with TA rapidly increasing to ∼58.71 μg mL^−1^ within 24 h before plateauing, whereas GHT-5 showed a slow and sustained release, reaching only ∼11.06 μg mL^−1^ at 24 h (Fig. S9A). Consistently, GHS-5 showed lower cell viability than GHT-5 (Fig. S9B-C), attributable to its burst-release behavior. Together, these results demonstrate that the TAWS strategy enables controlled TA incorporation, yielding a biocompatible release profile suitable for functional GBR membrane development.

Collectively, these results justify the selection of TAWS parameters (20 mm min^−1^, 0.5 h, 5 % TA), which achieve an optimal balance between efficient chain alignment, stable TA-mediated network locking, and maximal mechanical reinforcement without compromising structural integrity or biological performance.

### 3.3 Barrier Function and Stem Cell Recruitment effect

**Fig. 4:**
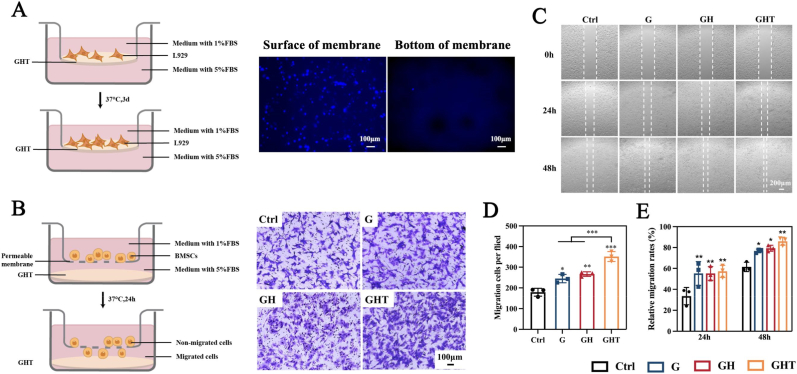
Barrier Function and Stem Cell Recruitment. (A) Schematic diagram of barrier function evaluation and images of L929 fibroblasts seeded on GHT membrane with cell nuclei stained by DAPI (blue). (B) The transwell assay of BMSCs induced by extracts from different hydrogels and the quantification analysis of cell migration. (C) Representative images of the wound healing capacities of BMSCs after coculturing with different hydrogels for the indicated time periods. Quantitative analysis of (D) transwell migration and (E) wound healing assays. (n = 3 for each group) Data are means ± SD; ∗*p* < 0.05, ∗∗*p* < 0.01, ∗∗∗*p* < 0.001.

A primary function of GBR membranes is to prevent soft tissue invasion and maintain a space for osteogenesis. To evaluate the barrier function of the fabricated GHT hydrogel, a transwell assay was performed. Results revealed dense fibroblast colonization on the hydrogel surface while the bottom of the hydrogel exhibited minimal cell adhesion (Fig. 4A). This marked reduction in cell transmigration confirms the GHT hydrogel's ability to effectively inhibit soft tissue infiltration, thereby supporting its potential as a functional GBR membrane.

Besides preventing soft tissue invasion, the capacity of GBR membranes to recruit endogenous stem cells is critical for successful bone regeneration. We therefore assessed the bone marrow stromal cells (BMSCs) recruitment potential of the GHT hydrogel using transwell migration and wound healing assays, comparing it to GelMA (G) and GelMA-HAp (GH) hydrogels. Transwell assays revealed significantly greater BMSCs migration toward GHT extracts than toward control, G, or GH extracts (Fig. 4B). This enhanced chemotaxis likely arises from synergistic effects: the GelMA's RGD motifs facilitate cell adhesion while TA promotes the recruitment of BMSCs [79,80]. Wound healing assays further demonstrated accelerated gap closure in GHT-stimulated groups. After 24 h and 48 h, GHT-treated cells exhibited significantly reduced gap widths compared to control, G, and GH groups (Fig. 4C). Semiquantitative analysis confirmed both increased migrated cell numbers (Fig. 4D) and decreased migration distances (Fig. 4E) in the GHT group, demonstrating nearly double the migration capacity of controls. Collectively, these findings indicate that the GHT hydrogel greatly accelerates BMSCs migration, facilitating stem cell recruitment to facilitate bone regeneration.

### 3.4 ROS scavenging ability of GHT hydrogel membranes in vitro

**Fig. 5:**
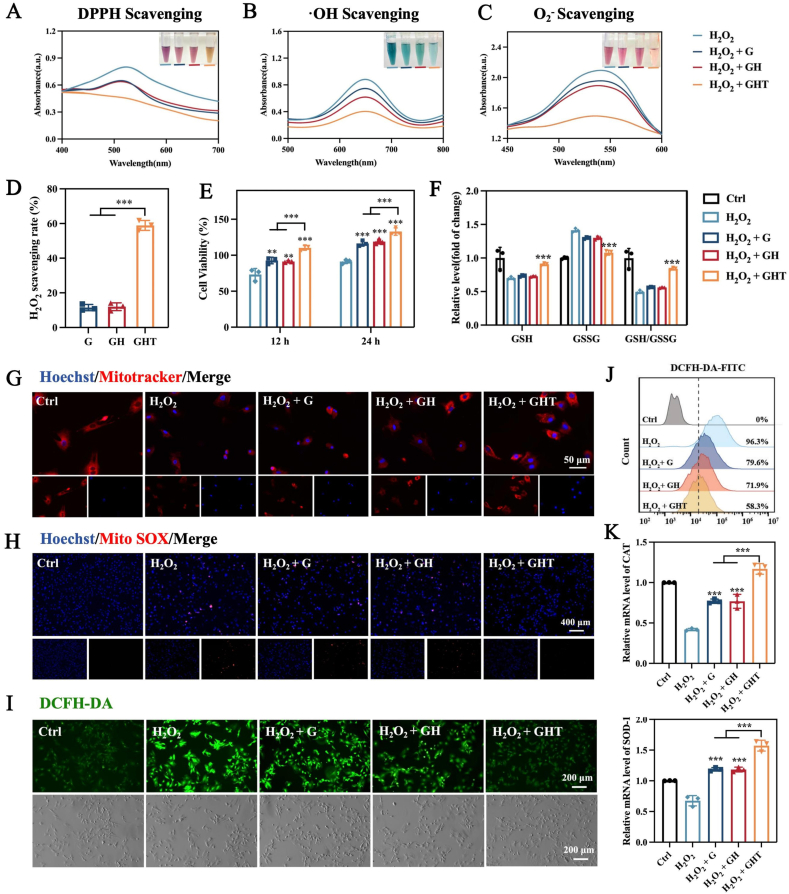
Antioxidant ability and intracellular ROS scavenging activity. (A–D) Scavenging of DPPH, ·OH, O_2_^−^and H_2_O_2_ by GHT hydrogel. (E) CCK-8 assays of BMSCs coculturing with the extracts from different hydrogels for 12 and 24 h with H_2_O_2_ stimulation. (F) The levels of reduced (GSH) and oxidized (GSSG) glutathione in BMSCs. (G) MitoTracker staining and (H) mtROS staining. (I) ROS staining, (J) FCM of DCFH-DA and the corresponding mean fluorescence intensity. (K) CAT and SOD-1 relative mRNA expressions after treatment with the extracts from different hydrogels under H_2_O_2_ stimulation. (n = 3 for each group) Data are means ± SD; ∗*p* < 0.05, ∗∗*p* < 0.01, ∗∗∗*p* < 0.001.

Excessive accumulation of reactive oxygen species (ROS) in the pathophysiological microenvironment of bone injuries induces cellular oxidative stress, impairing cellular function and significantly impeding bone repair [81,82]. Consequently, scavenging excess ROS is critical for successful regeneration. Tannic acid (TA) can act as a phenolic hydrogen donor, neutralizing free radicals. DPPH, hydroxyl radical (·OH), and superoxide (O_2_^−^) scavenging assays revealed that the GHT hydrogel exhibited significantly stronger radical-scavenging activity compared to the H_2_O_2_ group, while the G and GH hydrogels demonstrated moderate antioxidant effects, likely attributable to the inherent properties of GelMA (Fig. 5A–D,S10). Subsequent CCK-8 assays revealed that H_2_O_2_ exposure markedly reduced cell viability in the H_2_O_2_, H_2_O_2_ + G, and H_2_O_2_ + GH groups. In contrast, the H_2_O_2_ + GHT group effectively reversed this cytotoxicity, maintaining cell viability at levels comparable to the untreated control (Fig. 5E), indicating a strong protective effect against oxidative damage.

To further investigate intracellular oxidative stress, the levels of reduced (GSH) and oxidized glutathione (GSSG) in BMSCs were measured. Oxidative stress typically results in the oxidation of GSH to GSSG, decreasing the GSH/GSSG ratio. Notably, the H_2_O_2_ + GHT group maintained elevated GSH levels and a significantly higher GSH/GSSG ratio, indicating restoration of intracellular redox balance under oxidative stress (Fig. 5F). Mitochondrial integrity under oxidative stress was assessed using MitoTracker Deep Red FM staining (Fig. 5G). Cells in the H_2_O_2_ group exhibited reduced mitochondrial fluorescence, indicating impaired mitochondrial function, whereas cells in the H_2_O_2_ + GHT group showed restored fluorescence intensity, suggesting mitochondrial protection. Similarly, MitoSOX staining (Fig. 5H) revealed that GHT hydrogel extract treatment markedly decreased mitochondrial superoxide (mtROS) levels relative to the H_2_O_2_ group. Intracellular ROS levels were also quantified using the DCFH-DA fluorescent probe (Fig. 5I and J). Upon H_2_O_2_ stimulation, BMSCs displayed strong green fluorescence, indicative of elevated ROS. While the H_2_O_2_ + G and H_2_O_2_ + GH groups moderately reduced fluorescence intensity, the H_2_O_2_ + GHT group showed a substantial attenuation of ROS fluorescence. Flow cytometry analysis confirmed these findings, showing significantly lower fluorescence in the H_2_O_2_ + GHT group compared to the H_2_O_2_ control, with the H_2_O_2_ + G and H_2_O_2_ + GH groups displaying intermediate effects. To explore the molecular basis of antioxidant defense, the mRNA expression levels of antioxidant enzymes CAT and SOD-1 were measured by RT-qPCR (Fig. 5K). Consistently, the H_2_O_2_ + GHT group significantly upregulated CAT and SOD-1 expression, further supporting its role in enhancing intracellular antioxidant responses.

Collectively, these results demonstrate that the GHT hydrogel membrane effectively reverses H_2_O_2_-induced cellular damage by scavenging ROS and enhancing intracellular antioxidant defenses, thereby restoring redox homeostasis in the cellular microenvironment.

### 3.5 Assessment of in vitro osteogenic differentiation

Excessive accumulation of reactive oxygen species (ROS) at the injury site can lead to oxidative stress, disrupting key signaling pathways and gene expression profiles essential for osteogenesis, ultimately impairing bone regeneration [[83], [84], [85]]. Given the demonstrated ROS-scavenging capacity of the GHT membrane, we next investigated its ability to support osteogenic differentiation of BMSCs under oxidative stress conditions.

**Fig. 6:**
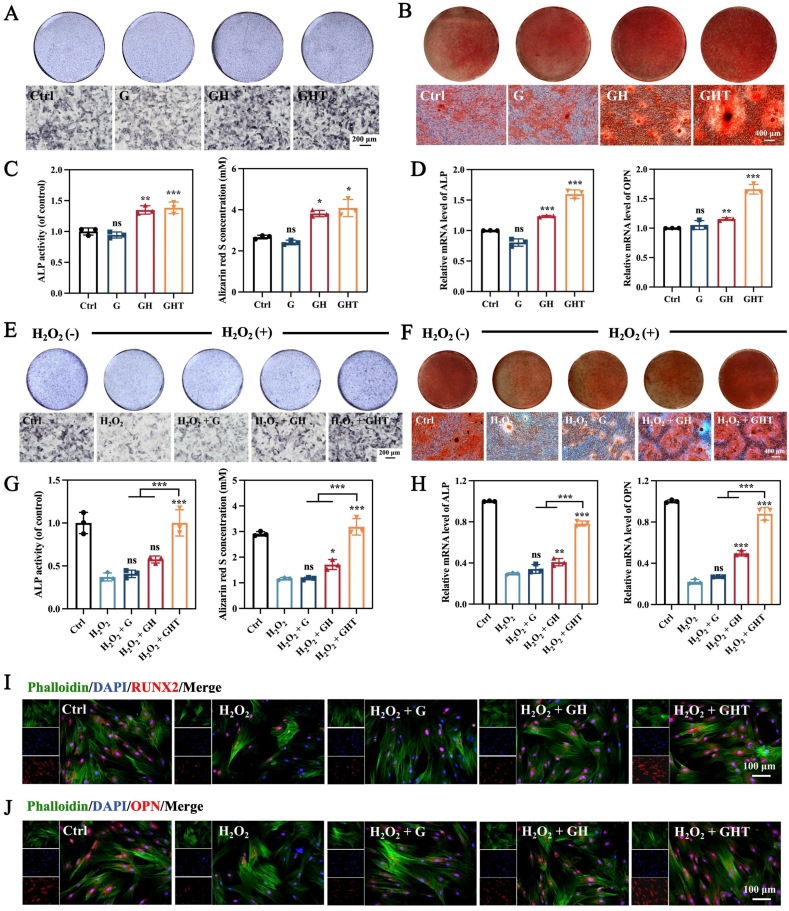
GHT hydrogels enhance osteogenic differentiation in vitro. (A) ALP staining of BMSCs after 7 days of induction. (B) ARS staining (day 21) of BMSCs. (C) Quantification of ALP activity (day 7) and ARS mineralization (day 21). (D) Relative mRNA expression of ALP and OPN (day 7). (E) ALP staining (day 7) and (F) ARS staining (day 21) of BMSCs under H_2_O_2_-induced oxidative stress. (G) Quantitative analysis of ALP activity (day 7) and ARS mineralization (day 21) under oxidative conditions. (H) Relative mRNA expression of ALP and OPN (day 7) with H_2_O_2_ stimulation. (I–J) Immunofluorescence staining of RUNX2 and OPN (day 7) under H_2_O_2_ exposure. ‘Control’ in 6C,G refers to untreated BMSCs without hydrogel extract treatment and H_2_O_2_ stimulation, with its activity set as 1.0 for normalization. (n = 3 for each group) Data are means ± SD; ∗*p* < 0.05, ∗∗*p* < 0.01, ∗∗∗*p* < 0.001.

To evaluate the influence of GHT hydrogels on osteogenic differentiation and their protective effects on stem cell function, we assessed the osteogenic potential of BMSCs cultured with or without H_2_O_2_ stimulation. Initially, osteogenic differentiation was examined under normal conditions (i.e., without H_2_O_2_ exposure) using alkaline phosphatase (ALP) and Alizarin Red S (ARS) staining to assess early and late osteogenic markers, respectively. After 7 days of osteogenic induction, ALP staining revealed a significantly greater staining area and intensity in both GH and GHT groups compared to the control and G groups, indicating enhanced early-stage differentiation (Fig. 6A–C). Similarly, after 21 days of induction, ARS staining and its quantitative analysis demonstrated increased calcium nodule formation in the GH and GHT groups (Fig. 6B and C). These findings are further supported by the upregulated expression of osteogenic genes, including ALP and osteopontin (OPN), and are primarily attributed to the osteoinductive effect of the incorporated HAp-NWs.

The osteogenic differentiation of BMSCs was further evaluated under oxidative stress conditions induced by H_2_O_2_. After 7 days of osteogenic induction, BMSCs exposed to H_2_O_2_ exhibited a 2.53-fold reduction in ALP activity compared to those cultured under normal conditions. Notably, treatment with GHT hydrogel extracts effectively restored ALP expression to levels comparable to the unstressed control group (Fig. 6E–G). At the later stage of differentiation (day 21), H_2_O_2_ exposure markedly suppressed calcium nodule formation, as evidenced by diminished ARS staining (Fig. 6F). In contrast, the GHT hydrogel significantly enhanced extracellular matrix mineralization under the same oxidative conditions, indicating successful osteogenic differentiation and mineral deposition by BMSCs (Fig. 6F and G). Further assessment of osteogenesis-related markers by RT-qPCR and immunofluorescence (IF) confirmed these findings. Gene expression levels of ALP and osteopontin (OPN) were elevated by 2.65-fold and 4.18-fold in the H_2_O_2_ + GHT group, respectively, compared to the H_2_O_2_ group (Fig. 6H). Similarly, protein expression of RUNX2 and OPN was significantly upregulated in the H_2_O_2_ + GHT group relative to the H_2_O_2_, H_2_O_2_ + G, and H_2_O_2_ + GH groups (Fig. 6I and J).

Collectively, these results indicate that GHT hydrogel extracts not only mitigate the detrimental effects of oxidative stress but also actively promote osteogenic differentiation. In summary, while excessive ROS impairs stem cell-mediated bone formation, the GHT hydrogel membrane protects BMSCs from ROS-induced damage and simultaneously exerts intrinsic osteoinductive effects, thereby rescuing and enhancing bone regeneration.

### 3.6 In vitro immunoregulation

Macrophages are key immune regulators in the bone regeneration microenvironment, where their phenotypic polarization plays a critical role in modulating inflammation and tissue repair [16]. To investigate the immunomodulatory effects of the GHT hydrogel membrane, RAW264.7 macrophages were used as a model system. Cells were first stimulated with 200 ng mL^−1^ LPS for 24 h to induce a pro-inflammatory (M1) phenotype, followed by a 6-h treatment with hydrogel extracts from each experimental group.

**Fig. 7:**
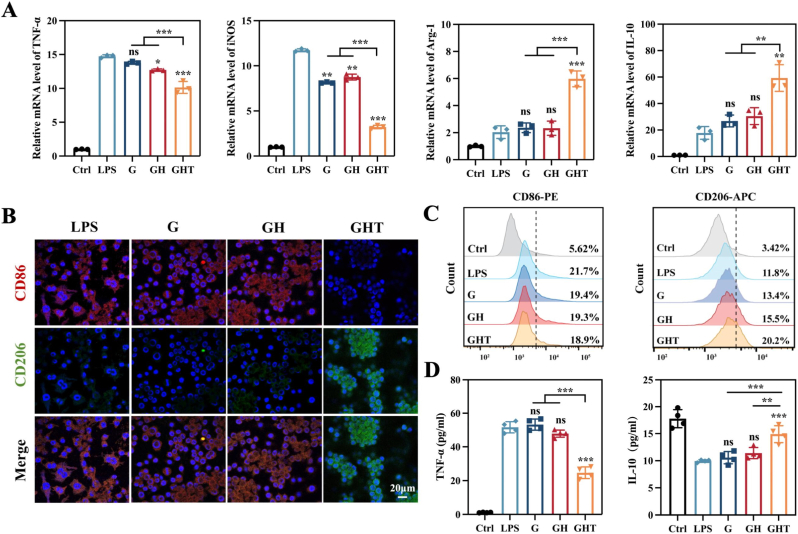
Regulation of macrophage polarization. (A) mRNA expressions of TNF-α, iNOS, IL-10 and Arg-1 (n = 3), (B) IF staining and (C) FCM of CD86 and CD206, and (D) ELISA of TNF-α and IL-10 in RAW264.7 cells when coculturing with extracts from different hydrogels for 24 h after LPS stimulation (n = 4). Data are means ± SD; ns: No significant differences, ∗*p* < 0.05, ∗∗*p* < 0.01, ∗∗∗*p* < 0.001.

Subsequent RNA analysis evaluated the expression of key inflammatory markers (TNF-α and iNOS for M1 phenotype, Arg-1 and IL-10 for M2 phenotype), with glyceraldehyde-3-phosphate dehydrogenase (GAPDH) used as the housekeeping gene (internal reference) for data normalization. The G and GH groups showed no significant modulation of macrophage polarization. In contrast, the GHT hydrogel significantly downregulated the expression of M1 markers: TNF-α and iNOS mRNA levels were reduced to 68.8 % and 27.4 % of the LPS-stimulated levels, respectively. Simultaneously, GHT treatment markedly upregulated M2-associated marker, with Arg-1 and IL-10 expression increasing by 2.94- and 3.33-fold respectively (Fig. 7A). Immunofluorescence (IF) staining confirmed these findings: CD86 (M1 marker) expression was substantially reduced, while CD206 (M2 marker) expression was significantly enhanced in GHT-treated macrophages (Fig. 7B). These observations were further supported by flow cytometry analysis (Fig. 7C and S11). Additionally, ELISA results showed a decrease in TNF-α secretion and a corresponding increase in IL-10 production in the GHT group, indicating a phenotypic shift from pro-inflammatory M1 to pro-regenerative M2 macrophages. No such shift was observed in the G or GH groups (Fig. 7D).

In conclusion, TA molecules released from the GHT hydrogel effectively overcame the immunomodulatory limitations of G and GH membranes, promoting a favorable macrophage polarization from M1 to M2. This phenotypic shift is expected to contribute to a coordinated, pro-regenerative bone healing microenvironment.

### 3.7 Bone regeneration in vivo

**Fig. 8:**
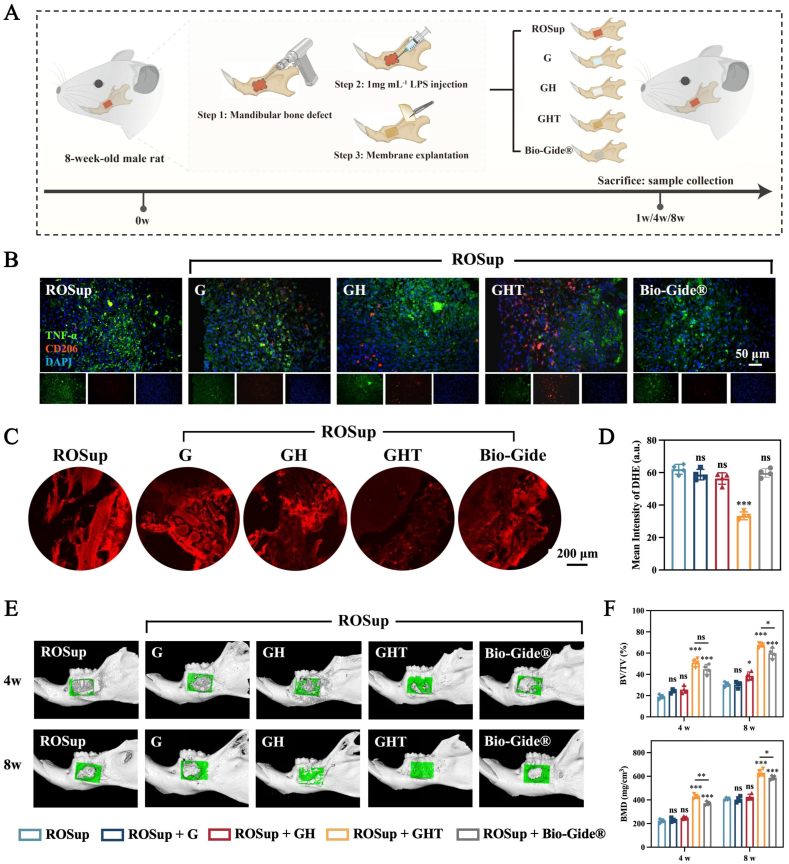
GHT hydrogels promote mandibular bone regeneration in vivo. (A) Schematic of the treatment schedule in rats. ROSup means ROS raised up after LPS stimulation. (B) IF staining images of TNF-α and CD206 at week 1 after operation. (C) Fluorescence staining images and (D) quantitative analysis of dihydroethidium (DHE) at week 1 after operation. (E) Reconstructed micro-CT images of mandibular bone defects at 4 and 8 weeks post-operation. (F) Quantitative analysis of BMD and BV/TV. (n = 4 for each group) Data are means ± SD; ns: No significant differences, ∗*p* < 0.05, ∗∗*p* < 0.01, ∗∗∗*p* < 0.001.

To further evaluate the regenerative potential of GHT hydrogel membranes in vivo, we established a LPS-induced mandibular bone defect model (5 × 4 × 1 mm^3^) in rats (Fig. S12). Defect regions were treated with 1 mg mL^−1^ LPS to create a reactive oxygen species (ROS)-enhanced microenvironment (designated ROSup) [81,86], then covered with G, GH, GHT or Bio-Gide® membrane (Fig. 8A). Dihydroethidium (DHE) and Immunofluorescence (IF) staining of TNF-α (M1 marker) and CD206 (M2 marker) confirmed significantly elevated levels of ROS and inflammation in ROSup defects at 1 week post-implantation compared to non-inflammatory controls (Fig. S13), validating successful model establishment. This ROSup mandibular defect recapitulates the pathological oxidative microenvironment of clinical inflammatory conditions such as periodontitis and periapical osteomyelitis, providing a physiologically relevant platform for evaluating barrier membrane functionality.

The in vivo anti-inflammatory and ROS-scavenging capacity of the GHT hydrogel membrane was first evaluated. As shown in Fig. 8B, IF staining and quantitative analysis of TNF-α (M1 marker) and CD206 (M2 marker) revealed that GHT membrane effectively attenuated pro-inflammatory responses and promoted M2 macrophage polarization, while Bio-Gide® showed limited immunomodulatory effects. Consistently, DHE fluorescence intensity in the GHT-treated group was significantly reduced to 53.76 % of that in the ROSup group, indicating a pronounced antioxidant effect. In contrast, no significant differences in ROS levels were observed among the G, GH, Bio-Gide®, and ROSup groups (Fig. 8C and D).

New bone formation at the defect site was evaluated by micro-CT, with quantitative morphometric analysis performed at 4 and 8 weeks post-surgery. In the 3D digital reconstruction images (new bone highlighted in green), the GH group exhibited limited bone formation originating from the defect margins, while the ROSup and G groups showed minimal regeneration, with clear defects remaining (Fig. 8G). Notably, GHT demonstrated a pronounced osteoinductive effect, achieving near-complete mandibular reconstruction by 8 weeks. The bone volume fraction (BV/TV) in the GHT group was 2.68-fold higher at week 4 and 2.21-fold higher at week 8 than in the ROSup group, and was also superior to that achieved with Bio-Gide® (Fig. 8H). Bone mineral density (BMD) measurements showed a similar trend, consistent with prior findings.

**Fig. 9:**
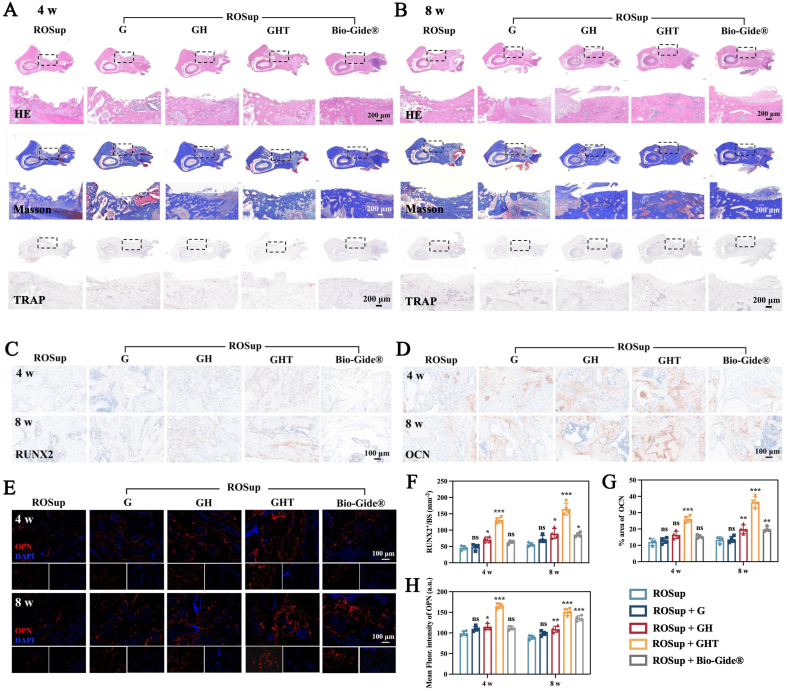
GHT hydrogels promote mandibular bone regeneration in vivo. H&E staining, Masson staining and TRAP staining of the bone defect area at (A) 4 and (B) 8 weeks post-operation. IHC staining of (C) RUNX2 and (D) OCN at 4 and 8 weeks post-operation. (E) IF images of OPN at 4 and 8 weeks post-operation. Quantitative analysis of (F,G) IHC (RUNX2, OCN) and (H) IF (OPN) at 4 and 8 weeks post-operation. (n = 4 for each group) Data are means ± SD; ns: No significant differences, ∗*p* < 0.05, ∗∗*p* < 0.01, ∗∗∗*p* < 0.001.

Consistent with micro-CT findings, histological analysis using H&E and Masson staining revealed that GHT exhibited a larger area of newly formed bone and higher bone tissue density than the ROSup, G, and GH groups at both 4 and 8 weeks post-operation. In addition, H&E sections at 4 and 8 weeks showed markedly reduced inflammatory cell infiltration in the GHT group compared with the other treatments, suggesting effective local microenvironment modulation and a lower likelihood of adverse chronic inflammation and fibrotic encapsulation (Fig. S14). TRAP staining revealed significantly fewer osteoclasts in GHT than in other groups, including Bio-Gide®, indicating suppressed osteoclast-mediated resorption and enhanced mineralization within the defect area (Fig. 9A and B).

To further evaluate in situ osteogenic differentiation, immunohistochemical (IHC) staining for RUNX2 and osteocalcin (OCN) and immunofluorescence (IF) staining for osteopontin (OPN) were performed. The number of RUNX2-positive cells (Fig. 9C–F) and positive area of OCN (Fig. 9D–G) were markedly higher in the GHT group than in other groups, including Bio-Gide®. Consistently, quantitative IF analysis showed that OPN expression in the GHT group was significantly upregulated relative to the ROSup group, increasing by 1.66- and 1.69-fold at 4 and 8 weeks, respectively, whereas Bio-Gide® showed less pronounced increases of approximately 1.13- and 1.52-fold (Fig. 9E–H). Together, these results indicate that GHT hydrogels effectively promote osteogenic differentiation and enhance bone regeneration in vivo, outperforming the Bio-Gide® membrane.

Finally, to assess potential systemic toxicity, major organs (heart, liver, spleen, lung, and kidney) were harvested at 4 weeks post-implantation, paraffin-embedded, and stained with H&E. Histological examination revealed no observable abnormalities or pathological changes in any of the groups, including GHT and Bio-Gide®, when compared to the untreated control (Fig. S15). In addition, considering that excessive TA exposure can induce extrinsic apoptosis through phenol–protein or phenol–lipid complex formation under oxidative conditions [87,88], local cytotoxicity was further assessed by TUNEL and cleaved caspase-3 staining. At 1 week post-implantation, few to no apoptotic cells were detected in the defect region of the GHT group, comparable to Bio-Gide® and slightly lower than in the ROSup, G, and GH groups subjected to LPS stimulation (Fig. S16). Collectively, these results indicate that TA release from GHT membranes remains within a safe range and does not induce local cytotoxicity.

## 4 Discussion

GelMA-based hydrogels are widely explored for bone regeneration due to their excellent biocompatibility and intrinsic cell-adhesive motifs, and the incorporation of hydroxyapatite (HAp) nanomaterials further enhance osteogenic differentiation and matrix mineralization [[89], [90], [91], [92], [93]]. Despite these biological advantages, GelMA hydrogels suffer from intrinsically poor mechanical strength and high swelling ratio arising from low crosslinking density and disordered polymer networks, which severely limits their ability to function as load-bearing barrier membranes in guided bone regeneration (GBR). In GBR, insufficient mechanical stability can lead to membrane collapse and soft-tissue invasion, ultimately compromising defect space maintenance and bone formation [31]. To overcome this critical limitation, we developed a tendon-inspired TAWS strategy that converts mechanically weak GelMA–HAp hydrogels into structurally robust membranes. Indeed, pristine GelMA–HAp (GH) hydrogels exhibited poor mechanical performance and limited physiological stability, with a low Young's modulus of 0.032 MPa and a high swelling ratio of ∼400 %. Following TAWS training, the resulting GHT hydrogels showed a dramatic enhancement in mechanical properties, including a 22.16-fold increase in Young's modulus to 0.716 MPa, together with markedly improved stability, as evidenced by a reduced swelling ratio of ∼100 %. Notably, TAWS-induced reinforcement was not strictly unidirectional: the Young's modulus measured perpendicular to the training axis was comparable to that along the tensile direction (Fig. S5), likely due to TA-mediated multivalent hydrogen bonding that stabilizes the GelMA network beyond simple chain alignment. Together with in vivo evidence that the GHT membrane maintained a 5 × 4 × 1 mm^3^ mandibular defect for 8 weeks without collapse or inward soft-tissue invasion (Fig. 8E and F and 9A,B), these results support sufficient multidirectional mechanical support for GBR. Collectively, these results demonstrate that the TAWS strategy effectively overcomes the mechanical limitations of natural hydrogels, substantially expanding their applicability as durable GBR membranes.

The mechanical reinforcement imparted by TAWS training arises from the synergistic effects of polymer chain alignment and strong GelMA–TA interactions, which together transform soft hydrogels into structurally robust networks. SEM analysis revealed that pristine GH hydrogels exhibited isotropic pore structures, whereas TAWS-trained GHT hydrogels displayed elongated, elliptical pores and a markedly denser microarchitecture, indicative of stretching-induced structural anisotropy. Consistently, SAXS showed isotropic scattering halos for GH hydrogels but elliptical scattering patterns for GHT hydrogels, directly confirming polymer chain alignment induced by wet stretching. Such aligned polymer networks are known to dissipate mechanical energy more efficiently and suppress crack initiation, thereby enhancing tensile strength and stiffness. Beyond chain alignment, TAWS training introduces strong noncovalent interactions between GelMA chains and TA molecules, which further stabilize the trained network. Acting as a multifunctional physical crosslinker, TA engages multiple GelMA chains through its abundant phenolic groups, effectively increasing the apparent crosslinking density and locking the aligned polymer configuration. This mechanism is supported by spectroscopic evidence: FTIR spectra exhibited a progressive red shift from GH to GHT hydrogels, indicating strengthened hydrogen bonding, while Raman spectroscopy revealed a pronounced blue shift in the C–O stretching vibration of TA with increasing TA concentration, consistent with extensive intermolecular interactions. Importantly, hydrogels trained in TA solutions exhibited an order-of-magnitude increase in Young's modulus and tensile strength compared with those trained in neutral salt solutions, underscoring that strong GelMA–TA interactions are essential for achieving durable mechanical reinforcement through TAWS training.

Beyond mechanical reinforcement, TA endows the TAWS-trained membrane with active biofunctionality by enabling sustained regulation of oxidative stress and inflammation within the defect microenvironment [94,95]. However, in most TA-based hydrogels or membranes, TA is introduced by simple immersion, resulting in the diffusive release of free TA that often leads to burst release and concentration-dependent cytotoxicity [[96], [97], [98]]. In contrast, the TAWS strategy embeds TA within the hydrogel network through strong molecular interactions, including hydrogen bonding and hydrophobic interactions, thereby coupling mechanical stabilization with controlled TA retention and release. Indeed, GelMA hydrogels immersed in TA solution without mechanical training exhibited rapid TA release accompanied by pronounced cytotoxicity, highlighting the necessity of TAWS for achieving safe and durable membrane functionalization (Fig. S9). Importantly, the mechanically stabilized and TA-retentive GHT membrane enabled sustained bioactivity in vitro, as evidenced by effective ROS scavenging, enhanced stem cell survival, proliferation, and osteogenic differentiation under oxidative stress, as well as promoted macrophage polarization toward the pro-regenerative M2 phenotype. In vivo, in a mandibular defect model characterized by elevated oxidative and inflammatory stress, implantation of the GHT membrane significantly reduced local ROS levels, attenuated inflammation, and increased bone volume fraction (BV/TV), new bone formation, and mineral density, outperforming the commercial GBR membrane BioGide®. Collectively, these results demonstrate that TAWS training not only preserves the mechanical integrity of the membrane but also provides a stable platform for sustained immunomodulation and redox regulation, thereby synergistically promoting bone regeneration under challenging inflammatory conditions.

Taken together, the TAWS strategy yields a multifunctional GBR membrane that integrates durable mechanical robustness with active immunomodulation and oxidative stress alleviation, thereby addressing multiple barriers to bone regeneration. In an inflammatory mandibular defect model, the GHT membrane outperformed the clinically used Bio-Gide® membrane, underscoring its therapeutic potential. Nevertheless, several challenges remain for clinical translation. Although short-term in vitro and in vivo studies demonstrate good biocompatibility, the long-term safety of TA, including systemic distribution, organ accumulation, and effects on mineral metabolism, requires further investigation. In addition, scaling TAWS to clinical-grade manufacturing will likely require automated and standardized processing to ensure reproducible mechanical training and TA-mediated reinforcement. Finally, further optimization of membrane thickness, mechanical anisotropy, and degradation kinetics will be necessary to meet the diverse requirements of clinical GBR. Future efforts will focus on long-term safety studies, scalable TAWS manufacturing, and refined material design to enable precise control over mechanical and degradation properties, thereby advancing clinical translation.

## 5 Conclusion

In conclusion, the tannic acid-assisted wet-stretching (TAWS) strategy successfully transforms mechanically weak natural hydrogels into robust and bioactive GBR membranes. By synergistically combining mechanical training-induced polymer alignment with TA-mediated chemical “locking”, the resulting membranes achieve exceptional long-term stability and strength. Beyond providing critical structural support, the intrinsically incorporated TA actively modulates the pathological microenvironment through potent ROS scavenging and immunomodulation. Ultimately, the GHT membrane significantly enhanced mandibular bone regeneration, underscoring its promising potential for engineering next-generation barrier membranes and translational applications.

## Funding

This work was supported by the 10.13039/501100001809National Natural Science Foundation of China (No. 82301081, No. 82470981, No. 82320108004), the 10.13039/501100012166National Key Research and Development Program of China (No. 2023YFC250630), the 10.13039/501100007129Natural Science Foundation of Shandong Province (No. 2023HWYQ-051), the 10.13039/501100004608Natural Science Foundation of Jiangsu Province (No. BK20230257), the 10.13039/100000084Construction Engineering Special Fund of “Taishan Scholars” of Shandong Province (NO. tsqn202306371, NO.tstp20250510and the National Clinical Key Specialty Construction Project (Periodontology, 2023).

## CRediT authorship contribution statement

**Jing Sun:** Investigation, Methodology, Software, Writing – original draft. **Xi Wang:** Methodology, Writing – review & editing. **Xiaoxue Wang:** Conceptualization, Writing – review & editing. **Wenhui Yu:** Methodology, Software. **Yang Yu:** Conceptualization, Supervision. **Shaohua Ge:** Conceptualization, Funding acquisition, Writing – review & editing. **Zheqin Dong:** Conceptualization, Funding acquisition, Supervision, Writing – original draft, Writing – review & editing.

## Declaration of competing interest

The authors declare that they have no known competing financial interests or personal relationships that could have appeared to influence the work reported in this paper.

## Data Availability

Data will be made available on request.
