# Supplementary material for: Tannic acid-assisted mechanical training transforms natural hydrogels into robust and bioactive membranes for guided bone regeneration
Source: Mater Today Bio. 2026 Feb 1;37:102863. doi: 10.1016/j.mtbio.2026.102863 (PMC12905784; doi:10.1016/j.mtbio.2026.102863)
Supplement: Multimedia component 1 [file mmc1.pdf]

Supporting Information

Tannic acid-assisted mechanical training transforms natural hydrogels into robust and bioactive membranes for guided bone regeneration

Jing Sun, Xi Wang, Xiaoxue Wang, Wenhui Yu, Yang Yu, Shaohua Ge*, and Zheqin Dong**

Department of Periodontology & Prosthodontics & Additive Manufacturing, School and Hospital of Stomatology, Shandong University & Shandong Key Laboratory of Oral Tissue Regeneration & Shandong Engineering Research Center of Dental Materials and Oral Tissue Regeneration & Shandong Provincial Clinical Research Center for Oral Diseases, Jinan, Shandong, China

***Corresponding Authors:**
Y. Yu, No.44-1 Wenhua Road West, 250012, Jinan, Shandong, China, Email:
Yuyang_dentist@sdu.edu.cn
S. Ge, No.44-1 Wenhua Road West, 250012, Jinan, Shandong, China, Email:
shaohuage@sdu.edu.cn
Z. Dong, No.44-1 Wenhua Road West, 250012, Jinan, Shandong, China, Email:
zheqindong@sdu.edu.cn

Table S1. Primer sets (Rat) used for quantitative real-time PCR.

RNA template	Forward primer (5′-3′)	Reverse primer (5′-3′)
GAPDH	TCTCTGCTCCTCCCTGTTCT	ATCCGTTCACACCGACCTTC
SOD-1	TACACAAGGCTGTACCACTGC	TCTTCATTTCCACCTTTGCCC
CAT	GGAGCTGGTAACCCAGTAGG	CCTTTGCCTTGGAGTATTTGGTA
ALP	GTTACAAGGTGGTGGACGGT	TAGTTCTGCTCATGGACGCC
OPN	GCTTGGCTTATGGACTGAGGTC	GCTTGGCTTATGGACTGAGGTC

Table S2. Primer sets (Mouse) used for quantitative real-time PCR.

RNA template	Forward primer (5′-3′)	Reverse primer (5′-3′)
GAPDH	GGGTCCCAGCTTAGGTTCAT	CCAATACGGCCAAATCCGTT
TNF-α	GCCTCCCTCTCATCAGTTCTA	GGCAGCCTTGTCCCTTG
iNOS	GAGACAGGGAAGTCTGAAGCAC	CCAGCAGTAGTTGCTCCTCTTC
Arg-1	CGCCTCAAATCCAGCTGTAAG	GGGCCACAATCCAGTCGTT
IL-10	CTGGACAACATACTGCTAACCG	GGGCATCACTTCTACCAGGTAA

Table S3. Sample abbreviations and descriptions.

Abbreviation	Description
Ctrl	Control group (untreated group, no hydrogel extracts or stimulators)
G	GelMA hydrogel
GH	GelMA-HAp composite hydrogel
GHS	GH hydrogel soaked in TA solution for 24 h
GHT	GH hydrogel subjected to mechanical training in TA solution
ROSup	ROS-enriched mandibular defect model
GHT-Salt	GH hydrogel subjected to mechanical training in saline (Na_2SO_4) for stability comparison
GHT-TA	GHT hydrogel (TAWS-trained) for stability comparison

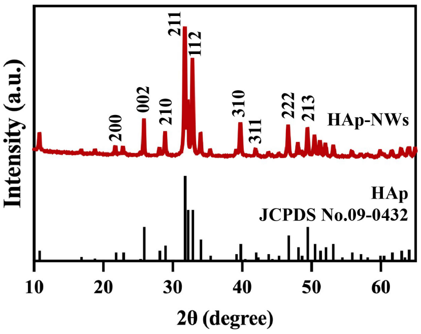

Fig. S1. XRD pattern of HAp nanowires (HAp-NWs).

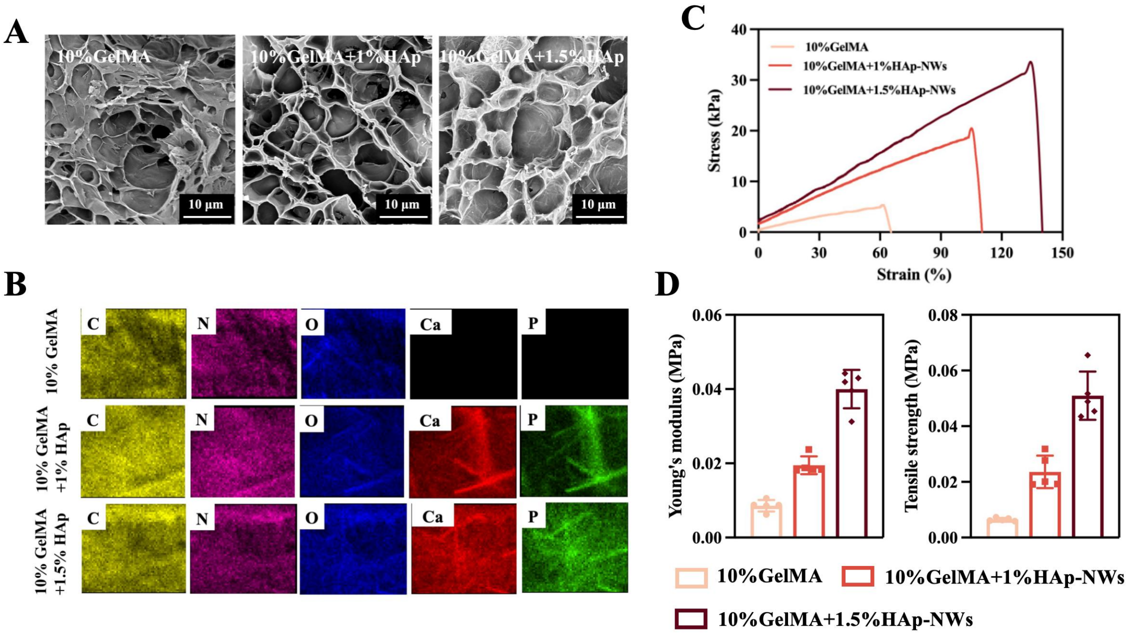

Fig. S2. Preparation and characterization of GH hydrogels. (A) SEM images and (B) EDS mapping of the GH hydrogels at different GelMA/HAp ratios. (C) Young's modulus and tensile strength of GH hydrogels at different GelMA/HAp ratios. (n = 5 for each group) Data are means ± SD.

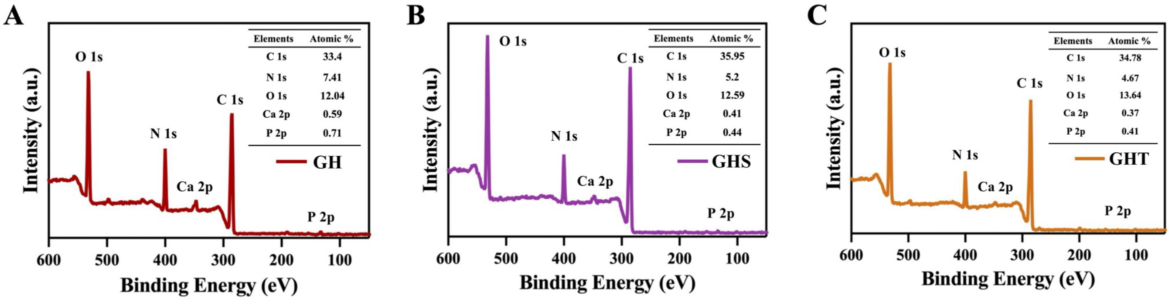

Fig. S3. XPS survey spectra of (A) GH, (B) GHS and (C) GHT.

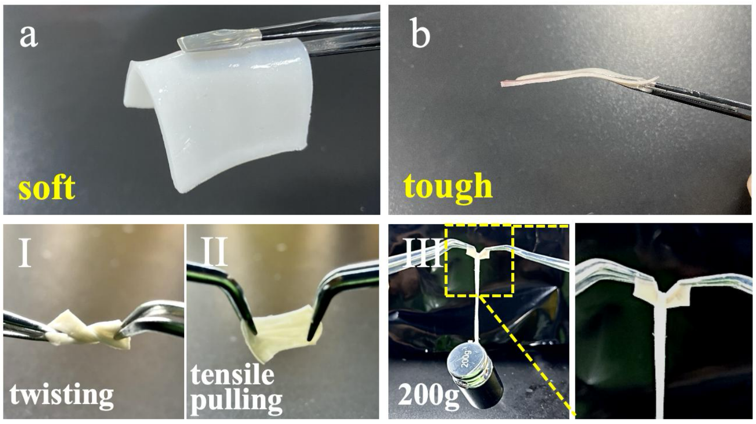

Fig. S4. Mechanical robustness of GHT hydrogels demonstrated through functional testing. GHT membranes maintained integrity during (I) twisting, (II) tensile pulling, and (III) suspension of a 200 g weight.

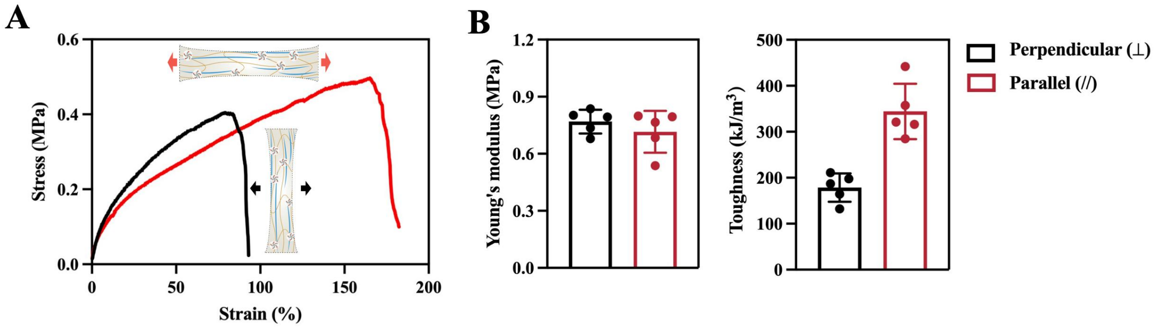

Fig. S5. Mechanical anisotropy of GHT hydrogels. (A) Representative stress-strain curves of GHT hydrogels tested along and perpendicular to the stretching direction. (B) Young's modulus and toughness measured in both orientations. (n = 5 for each group) Data are means ± SD.

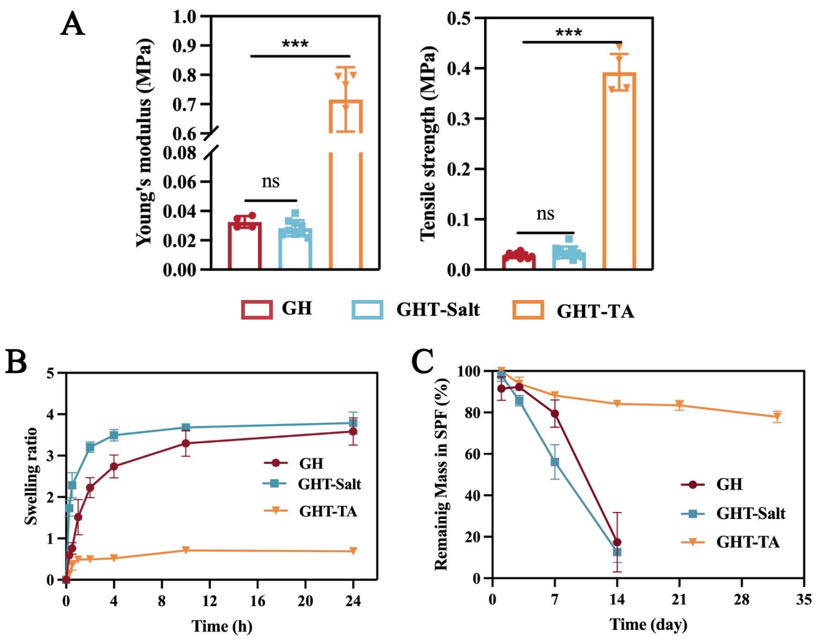

Fig. S6. Mechanical stability and anti-swelling property of GHT hydrogels. (A) Young's modulus and tensile strength of GH, GHT-Salt, and GHT-TA hydrogels after swelling equilibrium. (B) Swelling ratios and (C) degradation profiles in PBS for each group. (n = 5 for each group) Data are means ± SD. ns: No significant differences, $^{***}p < 0.001$.

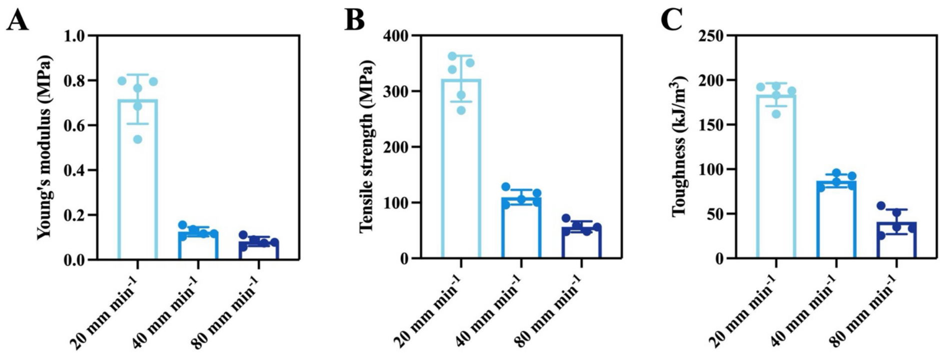

Fig. S7. Mechanical properties of GHT hydrogels as a function of training speeds. (n = 5 for each group)

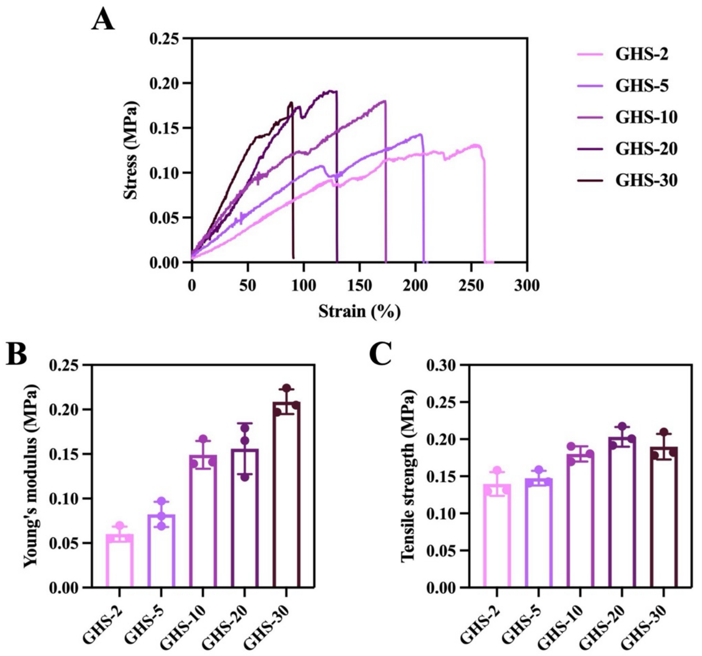

Fig. S8. Mechanical properties of TA-soaked (GHS) hydrogels at varying tannic acid concentrations. (A) Representative stress-strain curves of GHS hydrogels soaked in TA solutions of different concentrations. (B) Corresponding Young's modulus and tensile strength. (n = 3 for each group) Data are means ± SD.

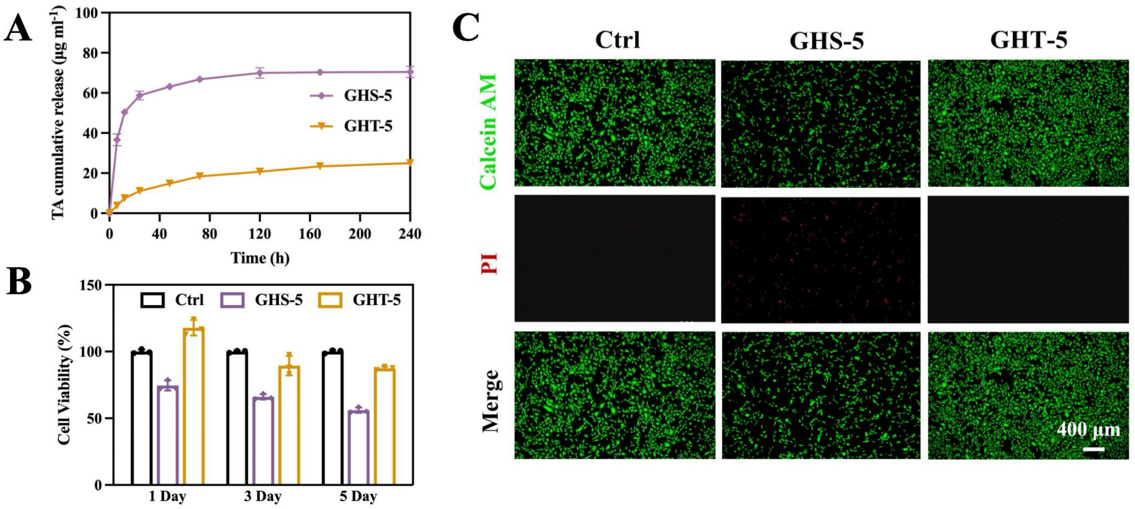

Fig. S9. Biocompatibility comparison between GHT and GHS hydrogels. (A) The release kinetics of TA in GHS and GHT hydrogels. (B) CCK-8 assay of cells cultured with extracts from GHS-5 and GHT-5 hydrogels for 1, 3, and 5 days. (B) Live/dead staining of cells after 48 h of exposure to the corresponding hydrogel extracts. (Calcein-AM for live cells, green; PI (propidium iodide) for dead cells, red). (n = 3 for each group) Data are means ± SD.

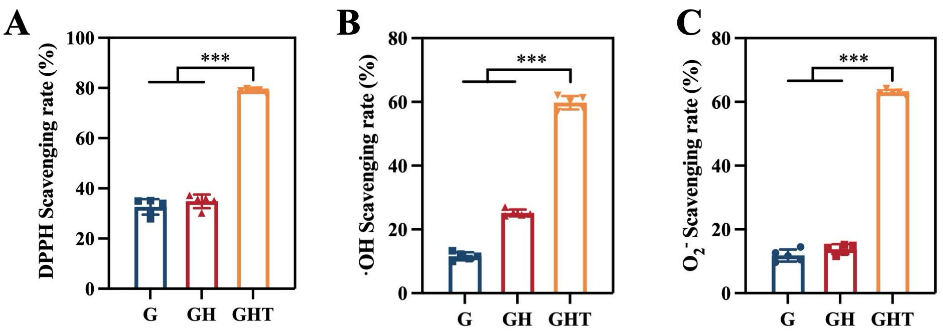

Fig. S10. Scavenging rate of (A) DPPH, (B) ·OH and (C) O_2^- by GHT hydrogel. (n = 5 for each group) Data are means ± SD. $^{***}p < 0.001$.

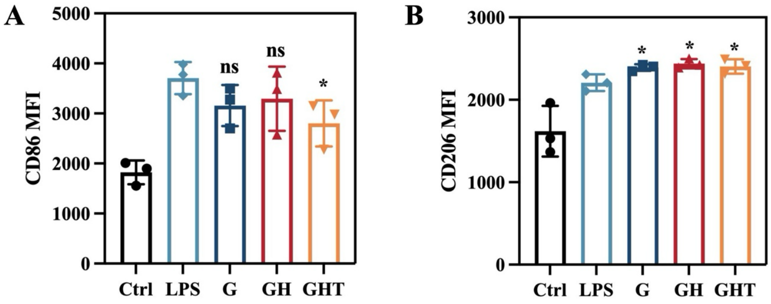

Fig. S11. Flow cytometric quantification of (A) $CD86^+$ and (B) $CD206^+$ macrophages. (n = 3 for each group) Data are means ± SD; ns: No significant differences, $^{*}p < 0.05$.

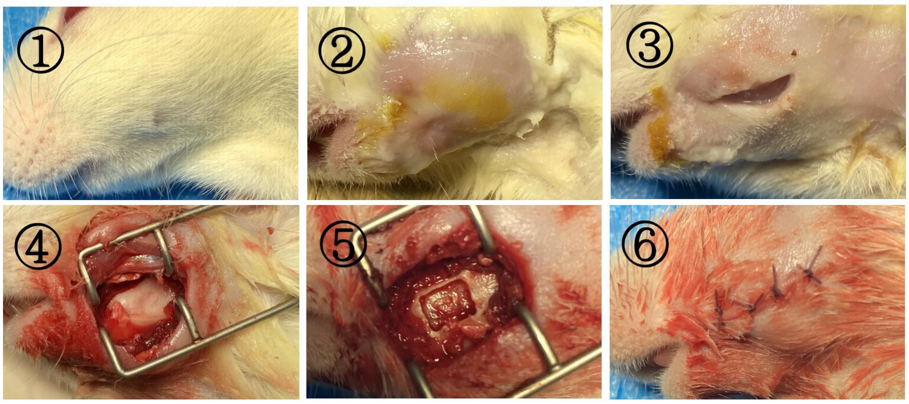

Fig. S12. Surgical creation of standardized mandibular bone defects ($5 \times 4 \times 1$ mm³) in 8-week-old male Wistar rats.

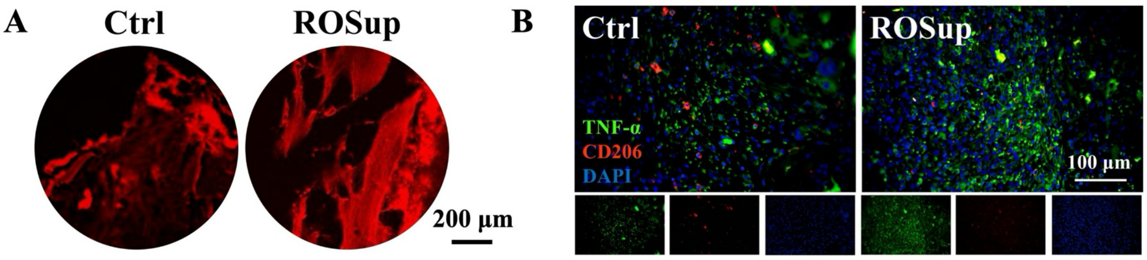

Fig. S13. Successful establishment of ROSup model in mandibular bone defects. (A) Fluorescence staining images of dihydroethidium (DHE) and (B) IF staining images of TNF-α and CD206 at week 1 after operation.

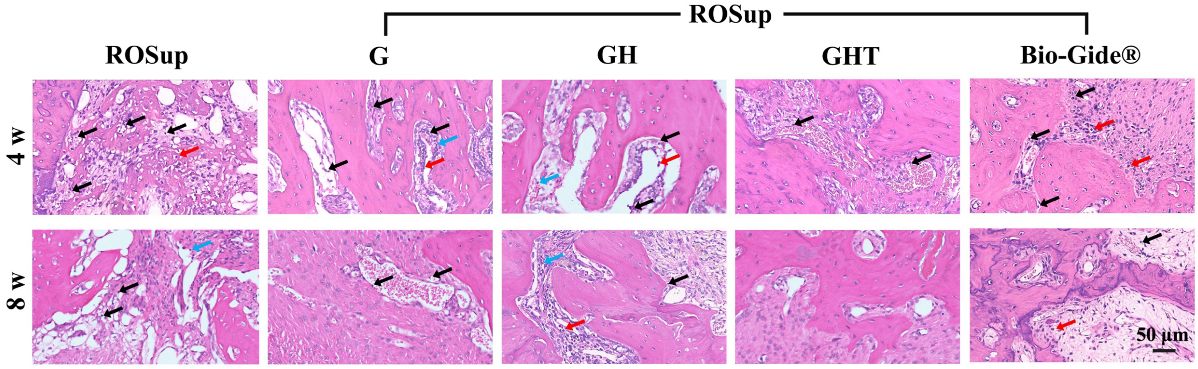

Fig. S14. H&E staining of mandibular defect regions at 4 and 8 week post-operation to evaluate inflammatory cell presence and infiltration (black arrow: neutrophil; blue arrow: plasma cell; red arrow: lymphocyte).

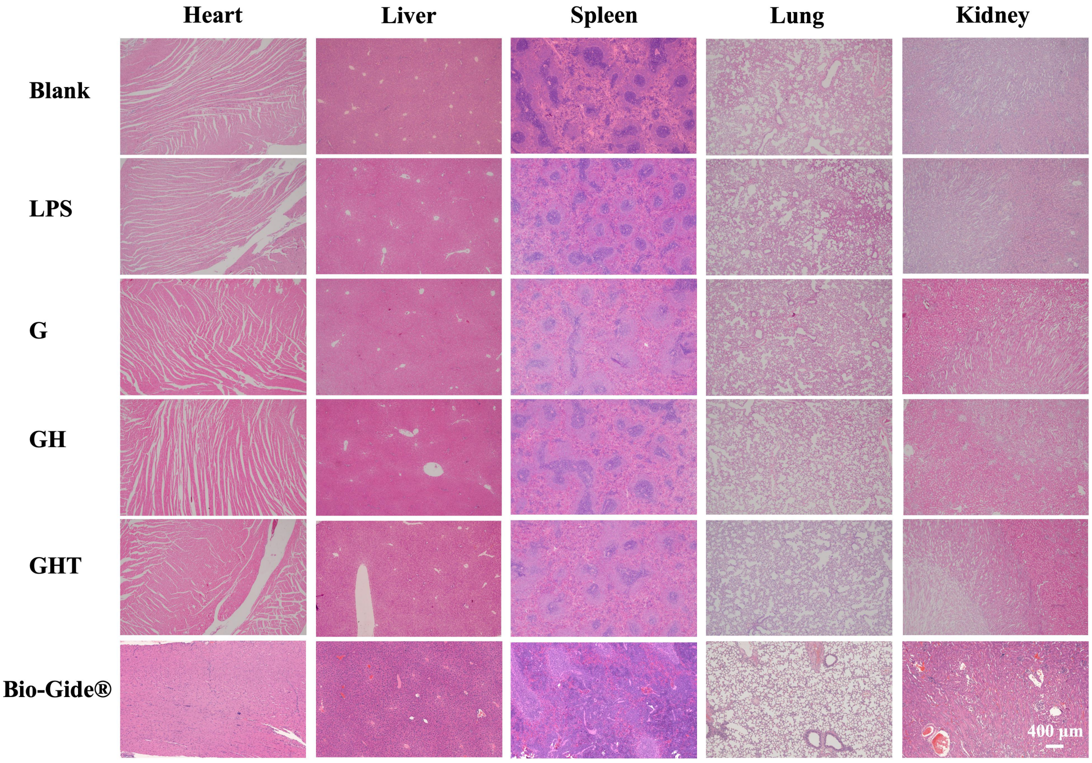

Fig. S15. In vivo biocompatibility assessment of hydrogels. H&E-stained sections of major organs (heart, liver, spleen, lung, and kidney) showed no observable pathological changes.

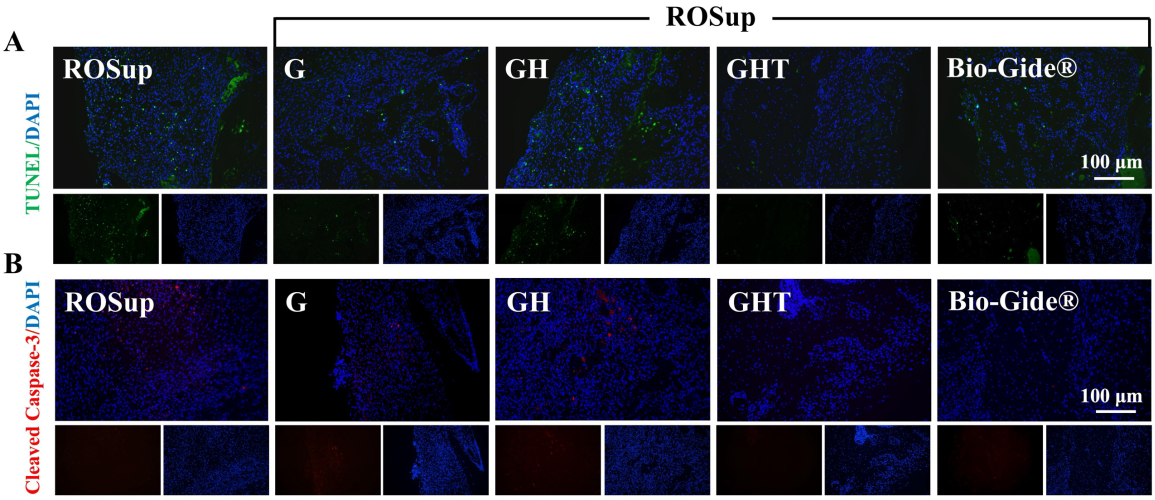

Fig. S16. Evaluation of cell apoptosis under oxidative stress by TUNEL staining and immunofluorescence (IF) of Cleaved Caspase-3.
